# Structural bioinformatics studies of glutamate transporters and their AlphaFold2 predicted water-soluble QTY variants and uncovering the natural mutations of L->Q, I->T, F->Y and Q->L, T->I and Y->F

**DOI:** 10.1371/journal.pone.0289644

**Published:** 2024-04-10

**Authors:** Alper Karagöl, Taner Karagöl, Eva Smorodina, Shuguang Zhang

**Affiliations:** 1 Istanbul University Istanbul Medical Faculty, Istanbul, Turkey; 2 Laboratory for Computational and Systems Immunology, Department of Immunology, University of Oslo, Oslo University Hospital, Oslo, Norway; 3 Laboratory of Molecular Architecture, Media Lab, Massachusetts Institute of Technology, Cambridge, MA, United States of America; Drexel University, UNITED STATES

## Abstract

Glutamate transporters play key roles in nervous physiology by modulating excitatory neurotransmitter levels, when malfunctioning, involving in a wide range of neurological and physiological disorders. However, integral transmembrane proteins including the glutamate transporters remain notoriously difficult to study, due to their localization within the cell membrane. Here we present the structural bioinformatics studies of glutamate transporters and their water-soluble variants generated through QTY-code, a protein design strategy based on systematic amino acid substitutions. These include 2 structures determined by X-ray crystallography, cryo-EM, and 6 predicted by AlphaFold2, and their predicted water-soluble QTY variants. In the native structures of glutamate transporters, transmembrane helices contain hydrophobic amino acids such as leucine (L), isoleucine (I), and phenylalanine (F). To design water-soluble variants, these hydrophobic amino acids are systematically replaced by hydrophilic amino acids, namely glutamine (Q), threonine (T) and tyrosine (Y). The QTY variants exhibited water-solubility, with four having identical isoelectric focusing points (pI) and the other four having very similar pI. We present the superposed structures of the native glutamate transporters and their water-soluble QTY variants. The superposed structures displayed remarkable similarity with RMSD 0.528Å-2.456Å, despite significant protein transmembrane sequence differences (41.1%—>53.8%). Additionally, we examined the differences of hydrophobicity patches between the native glutamate transporters and their QTY variants. Upon closer inspection, we discovered multiple natural variations of L->Q, I->T, F->Y and Q->L, T->I, Y->F in these transporters. Some of these natural variations were benign and the remaining were reported in specific neurological disorders. We further investigated the characteristics of hydrophobic to hydrophilic substitutions in glutamate transporters, utilizing variant analysis and evolutionary profiling. Our structural bioinformatics studies not only provided insight into the differences between the hydrophobic helices and hydrophilic helices in the glutamate transporters, but they are also expected to stimulate further study of other water-soluble transmembrane proteins.

## Introduction

Glutamate transporters are a class of membrane proteins that play a vital role in the central nervous system (CNS) by removing excess glutamate from the synapse, involving in critical mechanisms of synaptic plasticity, memory, and neuronal or glial cell death [1, 2]. Thus, the proper functioning of glutamate transporters is essential for neuronal physiology and healthy brain function [3]. Several subtypes of glutamate transporters are prevalent in peripheral organs, and their dysregulation has been associated with diverse types of tumors [4].

Vesicular glutamate transporters (VGLUTs) play a crucial role in the storage of glutamate, while the termination of glutamatergic signaling is predominantly mediated by the action of excitatory amino acid transporters (EAATs) located on the plasma membrane of astrocytes and neurons [3]. Consequently, alterations in the functions of these transporters have been associated with a range of psychiatric and neurophysiological disorders [1, 3, 5]. For instance, EAATs may be involved in the etiologies of Schizophrenia and affective disorders [6], and many other nervous system disorders [1, 3, 5]. VGLUTs may also play an important role in several neurophysiological disorders [1]. The functions of glutamate transporters extend well beyond the central nervous system, with a widespread presence in peripheral organs such as the heart, kidney, and liver [4]. Certain glutamate transporters also exhibit distributions in the placenta, emphasizing their roles in the healthy development of the human fetus [7]. Accordingly, the evidence for the roles of glutamate transporters in cancer biology is emerging as dysregulations can be seen in a range of tumor types [4].

While the glutamate transporters may present critical targets for therapeutics as some modulators are shown to have potential, current therapeutic options are limited because of poor efficacy [2]. However, it holds a significant premise to investigate new strategies to effectively regulate transporters. Nevertheless, unlike water-soluble proteins, the study and manipulation of the transporter proteins is a daunting task since they are embedded within a phospholipid bilayer membrane [8]. Due to their hydrophobic surface, detergents are required to isolate them, which is often unstable [8]. To overcome these challenges, we present an innovative alternative, as the QTY (Glutamine, Threonine, Tyrosine) code, which allows for the design of water-soluble domains without the use of detergents, instead through specific amino acid substitutions [9–11]. Alongside its promising role to develop new therapeutics while aiding researchers to generate effective therapeutic monoclonal antibodies, these soluble QTY variants of glutamate transporters may have several additional benefits; from designs of membrane proteins with improved properties; to potentially even the discovery of new functions.

We previously applied the QTY code to design a range of detergent-free transmembrane protein chemokine receptors and cytokine receptors and used conventional computing programs in this process [9–11]. The expressed and purified water-soluble variants exhibited the predicted characteristics and maintained their ligand-binding activity [9–14]. After the AlphaFold2 was released in July 2021, we immediately used AlphaFold2 to make QTY variant protein structure predictions and achieved improved results in less than an hour [15–18], compared to the previous method which took approximately 5 weeks per simulation [9–11]. Additionally, we developed a program and website for designing water-soluble QTY variants of membrane proteins [19]. The reverse QTY-code was recently described based on similar biochemical characteristics [20]. AlphaFold2 greatly accelerated research on predictions of protein structures with high accuracy, enabling the design of novel proteins, and the identification of new protein interactions and functions [21].

We hereby report using the combination of multiple approaches including glutamate transporter structural analysis, genomic variant analysis, and evolutionary conservation studies, we can significantly advance our understanding of protein structures and ultimately allow effective options for the fields of medical treatment and diagnosis. A large number of protein-coding gene variants found in populations may provide researchers with a valuable tool. Such variant analysis is essential for drug design, as it enables the identification of amino acid residues crucial for a protein’s activity or those that may be targeted by inhibitors. Furthermore, using archives of the human genetic variations found in patient samples, such as ClinVar [22], we show the phenotypical effects of the variants. Insights gained from evolutionary conservation studies may further aid the protein design process. Particularly in the case of glutamate transporters since the structural mechanism of amino acid symport is evolutionarily conserved from archaea to humans [23].

Our findings here provide a comprehensive analysis of the glutamate transporters and their water-soluble QTY variants while demonstrating the viability of in silico tools to manipulate the characteristics of vital transmembrane proteins. By utilizing specific approaches to generate water-soluble variants of proteins including the QTY code, researchers may be able to develop more effective therapies and diagnostic tools for various disorders that caused by dysregulation of glutamate transporters.

## Methods

### Protein sequence alignments and other characteristics

The UniProt [24] website (https://www.uniprot.org) provides protein ID, entry name, description, and FASTA sequence information for each protein. The UniProt accession numbers for the EAATs 1–4, VGLUTs 1–3, and YLAT2 are P43003, P43004, P43005, P48664, Q9P2U7, Q9P2U8, Q8NDX2, and Q92536, respectively. The sequences were available from UniProt [24]. The QTY code was applied to transmembrane alpha-helices of each protein sequence, using the topological information and cellular locations of the mature proteins that were also derived from UniProt database [24]. The membrane topology and other sequence features then visualized by plots generated using Protter web application (https://wlab.ethz.ch/protter/) [25]. The obtained secondary structures and sequence alignments visualized using the 2dSS web server (http://genome.lcqb.upmc.fr/2dss/) [26].

For comparing effects of the QTY code on the membrane spanning regions, transmembrane helix predictions for both native transporters and their QTY variants were carried out using TMHMM -2.0 [27, 28], based on a hidden Markov model. The molecular weights (MW) and isoelectric point (pI) values of the native transporters and their QTY variants were calculated using the Expasy website (https://web.expasy.org/compute_pi/) [29–31].

### AlphaFold2 predictions

The structure predictions of the QTY variants were performed using the AlphaFold2 [21, 32] program, which can be accessed at (https://github.com/sokrypton/ColabFold). The program was run on 2 x 20 Intel Xeon Gold 6248 cores with 384 GB of RAM and a Nvidia Volta V100 GPU, following the instructions provided on the website. The European Bioinformatics Institute (EBI) houses over 200 million AlphaFold2-predicted structures and can be found at (https://alphafold.ebi.ac.uk).

### Superposed structures

The experimentally-determined structures used in this study are EAA1 (PDB ID: 5LLM) [33] and EAA3 (PDB ID: 6X2Z) [23] that were obtained from the RCSB PDB database. (https://www.rcsb.org) [34]. The superposition of structures was performed for EAA1^Crystal^ *vs* EAA1^QTY^, and EAA3^CryoEM^ *vs* EAA3^QTY^.

The native structures of eight transporters and their QTY variants were predicted using AlphaFold2. The superposition of these structures was performed using PyMOL [35], which is available at (https://pymol.org/2/).

### Structure visualization

In the study, two software programs were utilized for structure visualization: PyMOL [35] (https://pymol.org/2/) and UCSF ChimeraX [36] (https://www.rbvi.ucsf.edu/chimerax/). PyMOL was used for the superposition of molecular models, whereas the representation of hydrophobicity models was accomplished utilizing ChimeraX. Additionally, the visualization of natural mutations of the QTY variants was also performed using the ChimeraX software.

### Data acquisition and variant analysis

Variants containing natural variations of QTY (L->Q, I->T, and F->Y) and reverse QTY (Q->L, T->I, and Y->F) submitted by large-scale sequencing projects obtained from the Genome Aggregation Database [37] (gnomAD v2.1.1, http://gnomad.broadinstitute.org/). QTY and reverse QTY mutations were analyzed using gnomAD browser, disease-associated variants from the ClinVar database [22] (https://www.ncbi.nlm.nih.gov/clinvar/) and *in silico* variant impact predictions from Polyphen-2 [38] (http://genetics.bwh.harvard.edu/pph2/) were listed, resulting in a final dataset of 95 missense protein variants. Identified secondary structures of native transporters were manually inspected and the transporter topology obtained through UniProt data later correlated with the missense amino acid variants.

### Building natural QTY and rQTY mutation libraries

PolyPhen-2 [38] (http://genetics.bwh.harvard.edu/pph2/) was used to predict the impact of the mutations on the protein function and structure. The input data for PolyPhen-2 analysis included all 19 amino acids substitutions possible to occur at the residue, which natural QTY or rQTY substitutions occurred. More than 1,800 potential variations analyzed, and the predicted effects were subsequently visualized using GNUPlot [39].

### Building mutation libraries for the TM regions of EAA1

We used Polyphen-2 [38] to predict the effects of all 19 amino acids substitutions at the residue of L, I, V, F amino acids in the TM α-helices of the EAA1 (total 97 amino acids), regardless of their occurrence in the population or nature. The predicted effects of 1,843 variations were plotted using GNUPlot [39] and L, I, V, F -> Q, T, Y substitutions compared with other amino acid substitutions.

### Evolutionary conservation profiles and analysis of sensitive domains

Mutation visualizations for glutamate transporters were accessed from PMut Repository [40] (https://mmb.irbbarcelona.org/PMut/). ConSurf server [41–46] (https://consurf.tau.ac.il/) used for generating evolutionary conservation profiles. The server ran with AlphaFold2 predicted native structures that were also used for RMSD calculations, and these structures were later complemented with SEQRES records. The.pdb files generated from AlphaFold2 did not contain the SEQRES sequences at the onset. The source sequences for the protein structures were derived from Uniprot in FASTA format. To translate and add the amino acid sequences to the.pdb files in the correct SEQRES format, visual basic for applications (VBA) scripting was utilized.

The conservation scores were computed using the Bayesian method, with the amino acid substitution model chosen based on the best fit. The default parameters were employed for homologues search, homologues thresholds and alignment, phylogeny, and conservation scores. The evolutionary conservation grades of each residue were visualized using the UCSF ChimeraX [36] software (https://www.rbvi.ucsf.edu/chimerax/). The conservation grades and residue exposure data obtained from the ConSurf server were complemented with secondary structure information and transporter topology. Per-residue helix and strand assignments of native glutamate transporters were deduced from the models available in the AlphaFold Database [21, 32], the algorithm for Defining the Secondary Structure of Proteins (DSSP) [47] were run using UCSF ChimeraX [36] (https://www.rbvi.ucsf.edu/chimerax/). The default energy cut off parameters of -0.5 kcal/mol, as recommended by Kabsch and Sander [47], were used for the calculations, minimum number of residues allowed in a helix or strand were also set to the default value of 3. These data were subsequently correlated with the predicted phenotypical and structural effects of the natural QTY (as well as rQTY) variants investigated in this study.

### AlphaFold2 predicted water-soluble QTY variants

The AlphaFold DB [21, 32] (https://alphafold.ebi.ac.uk), a database developed by DeepMind and the European Bioinformatics Institute (EMBL-EBI) at EMBL, serves as the repository for all AlphaFold2 predictions, with over 200 million protein structures. For more detailed information on the water-soluble QTY variants that are reported in this study, please go to the website: https://github.com/eva-smorodina/glut.

## Results and discussions

### Protein sequence alignments and other characteristics

**Fig 1:**
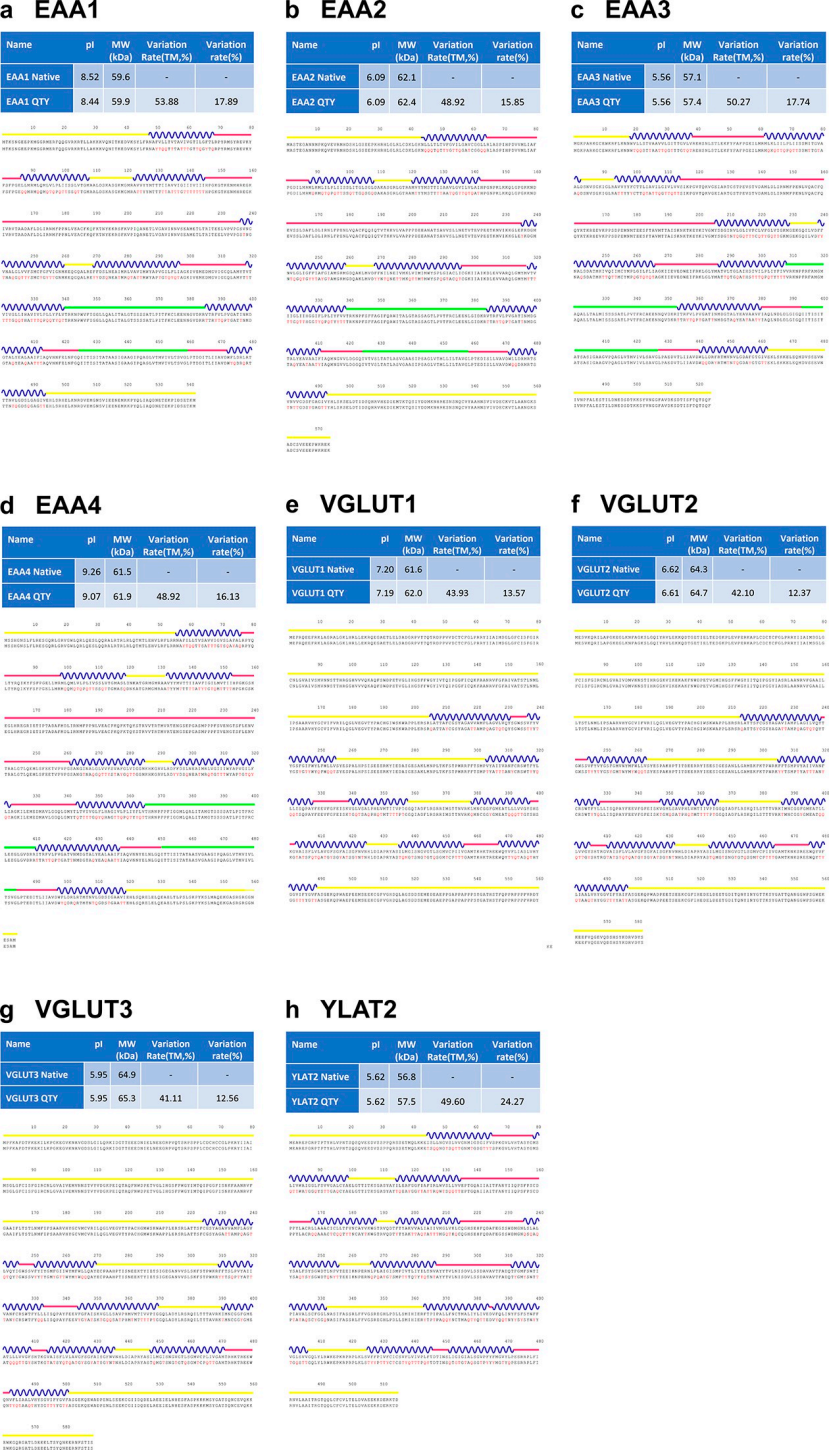
Sequence and protein alignments of the native and QTY variants of eight glutamate transporters. The alignments performed are as follows: **a** EAA1 *vs* EAA1^QTY^, **b** EAA2 *vs* EAA2^QTY^, **c** EAA3 *vs* EAA3^QTY^, **d** EAA4 *vs* EAA4^QTY^, **e** VGLUT1 *vs* VGLUT1^QTY^, **f** VGLUT2 *vs* VGLUT2^QTY^, **g** VGLUT3 *vs* VGLUT3^QTY^, and **h** YLAT2 *vs* YLAT2^QTY^. Molecular weight, isoelectric point (pI), total variation %, and transmembrane variation % are listed for both the natural and QTY variants. The TM alpha-helices (blue) are shown above the protein sequences. The QTY amino acid substitution changes are colored in red. Other color code: Yellow line, intracellular; Blue wave-transmembrane helices; Pinkish line, extracellular; Green line, peripheral domains and hairpin loops. Single letter abbreviations for the amino acid residues are A, Ala; C, Cys; D, Asp; E, Glu; F, Phe; G, Gly; H, His; I, lle; K, Lys; L, Leu; M, Met; N, Asn; P, Pro; Q, Gln; R, Arg; S, Ser; T, Thr; V, Val; W, Trp; and Y, Tyr.

The topological visualizations and predicted sequence features of EAATs and VGLUTs indicated that each transporter has an 8-transmembrane (TM) architecture, whereas the Y+L amino acid transporter-2 (YLAT2) has 12TM MFS-fold transporter topology (S2 Fig in S1 File) [23, 33, 48]. Contrary to VGLUTs topology, EAATs also has a larger extracellular loop between TM3 and TM4, which is absent in the structures determined by X-ray crystallography or cryo-EM methods [23, 33]. Meanwhile, VGLUTs have a larger portion of intracellular motifs than those in EAATs and YLAT2 (S2 Fig in S1 File). The isoelectric points (pIs) of the transporters varied between 9.26 for EAA4 and 5.56 for EAA3 (Fig 1 and S22 Fig in S1 File and Table 1).

**Table 1:** Characteristics of native glutamate transporters and their water-soluble QTY variants.

Name	RMSD	pI	MW (KD)	TM variation (%)	Overall variation (%)
EAA1 -		8.52	59.6	-	-
EAA1^QTY^	0.717Å	8.44	59.9	53.88	17.89
EAA2 -		6.09	62.1	-	-
EAA2^QTY^	0.948Å	6.09	62.4	48.92	15.85
EAA3 -		5.56	57.1	-	-
EAA3^QTY^	0.905Å	5.56	57.4	50.27	17.74
EAA4 -		9.26	61.5	-	-
EAA4^QTY^	0.796Å	9.07	61.9	48.92	16.13
VGLUT1 -		7.2	61.6	-	-
VGLUT1^QTY^	1.604Å	7.19	62	43.93	13.57
VGLUT2 -		6.62	64.3	-	-
VGLUT2^QTY^	0.971Å	6.61	64.7	42.1	12.37
VGLUT3 -		5.95	64.9	-	-
VGLUT3^QTY^	1.422Å	5.95	65.3	41.11	12.56
YLAT2 -		5.62	56.8	-	-
YLAT2^QTY^	0.528Å	5.62	57.5	49.6	24.27

Residue mean-square distance (RMSD) in Å, Isoelectric focusing (pI), Molecular weight (MW), Transmembrane (TM),— = not applicable. The internal and external loops have no changes, the overall changes are significant, and the TM changes are rather large.

The QTY (Glutamine, Threonine, Tyrosine) code substitute four hydrophobic amino acids (Leucine, Isoleucine, Valine, and Phenylalanine) with three neutral polar amino acids (Glutamine, Threonine, and Tyrosine) in transmembrane segments, reducing hydrophobicity. The 1.5Å electron density maps show very similar structures between leucine (L) *vs* glutamine (Q); isoleucine (I), valine (V) *vs* threonine (T); and phenylalanine (F) *vs* tyrosine (Y), leading to the implementation of the QTY code.

The QTY code results in significant substitutions in the transmembrane helices, ranging from 41% to 54% (Table 1). Despite the high substitution rate, the difference in molecular weight between the native and QTY variants is only a minimal amount, in the range of a few hundred Daltons (Da). This observation can be attributed to two factors. First, the substitution of the CH3- group (15Da) on leucine (L) and valine (V) with -OH groups (17Da) on glutamine (Q) and threonine (T) results in 2Da loss per substitution. Second, the addition of an OH- group occurs while the substitution of phenylalanine (F) to tyrosine (Y) takes place. The sum of these changes results in a minor effect on the molecular weights of the proteins (Table 1). Furthermore, previous experimental research demonstrated that QTY variants show remarkable thermostability [9, 10], despite the variants having a reduced number of aliphatic residues (A, L, V, I), resulting from the substitution of L with Q, and I as well as V with T. Additionally, the QTY substitutions does not introduce any charged residues into the protein, thus resulting in minimal changes of pIs, which could lead to non-specific interactions if changed.

### AlphaFold2 predictions

Understanding the 3D structure of transmembrane proteins is a crucial task, as it is key to understanding how they function, interact with other molecules, and can be targeted for therapeutic purposes. However, experimentally determining the structure of transmembrane proteins is a notoriously difficult process, owing to the hydrophobic nature of transmembrane proteins that require detergents to stabilize the membrane protein after isolating them from the cell membrane. From gene expression, and protein production, to selecting the appropriate detergent for maintaining stability, and avoiding irreversible aggregation, every step poses significant challenges [8]. Thus, the quantity of 3D structures experimentally determined for transmembrane proteins is significantly lag behind in comparison to that of water-soluble proteins. Consequently, Alphafold2 has a significant impact on the field of transmembrane protein research by providing researchers with accurate molecular structural models [21, 32].

In previous work, we used AlphaFold2 to predict the structures of water-soluble QTY variants of G protein-coupled receptors [15], glucose transporters [16], solute carrier transporters (SLC) [17], and potassium ion channels [18]. These predictions were in agreement with previously known experimentally-determined structures obtained through X-ray crystallography or cryo-EM methods. In this study, we also utilize AlphaFold2 to predict QTY variant and native transporters, as well as comparing them with two experimentally native determined structures.

### Superposition of native transporters and their water-soluble QTY variants

In our current study, the native transporter structures determined by cryo-EM, or X-ray crystallography were superimposed and compared to their QTY variants. The experimentally-determined structures used in this study are EAA1 (PDB ID: 5LLM) [33] and EAA3 (PDB ID: 6X2Z) [23], both obtained from RCSB PDB. The superposition of structures was performed for EAA1^Crystal^ *vs* EAA1^QTY^, and EAA3^CryoEM^ *vs* EAA3^QTY^.

**Fig 2:**
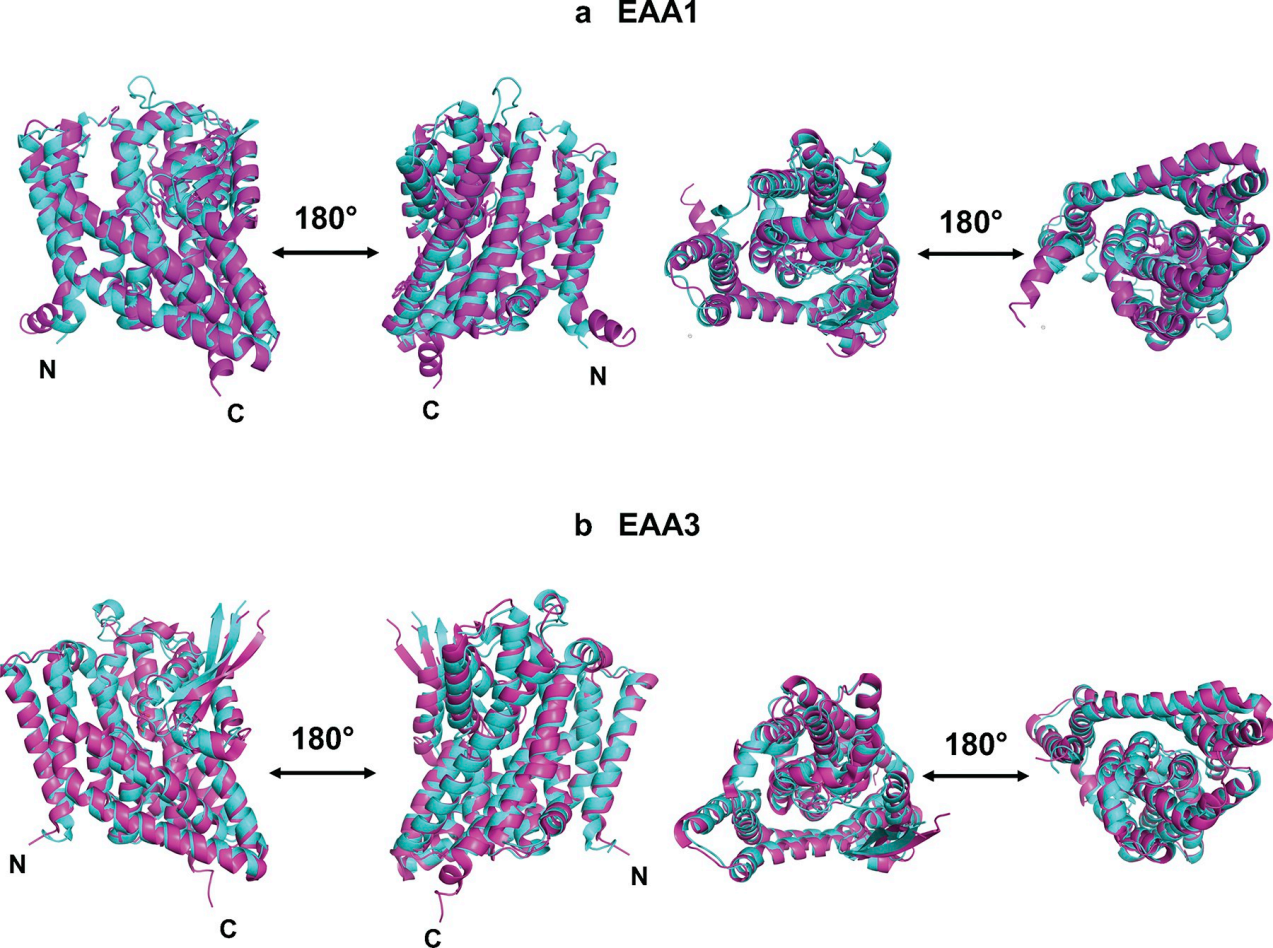
Superposed cryo-EM and crystal structures of EAA1^Crystal^ and EAA3^CryoEM^ with AlphaFold2 predicted QTY water-soluble variant and EAA1^QTY^ and EAA3^QTY^. The structures of native EAA1 (PDB ID: 5LLM, 3.25Å) and EAA3(PDB ID: 6X2Z, 3.03Å) are obtained from the Protein Data Bank. N- and C-termini are labelled. (**a**) The EAA1^Crystal^ (magenta) is superposed with AlphaFold2 predicted water-soluble variant EAA1^QTY^ (cyan). The RMSD is 1.729Å. (**b**) The Cryo-EM structure EAA3^CryoEM^ (magenta) is superposed with AlphaFold2 predicted water-soluble variant EAA3^QTY^ (cyan). The RMSD is 2.456Å. N- and C termini and large loops that are not resolved in the experimental structures are removed for clarity of direct comparisons.

The cryo-EM/crystal structures of native proteins and their AlphaFold2 predicted water-soluble QTY variants were superposed less than 2.5Å (Fig 2). Despite a high substitution rate of 54% in the transmembrane alpha-helices in the water-soluble QTY variants, their structures remain similar to the native structures, demonstrated by the root mean square deviation (RMSD). The RMSD values for EAA1^crystal^ vs EAA1^QTY^ were 1.729Å, and for EAA3^CryoEM^ vs EAA3^QTY^ were 2.456Å (Fig 2). The molecular structures, both experimentally determined and predicted by AlphaFold2, were found to superpose very well. Furthermore, the cryo-EM and crystal structures were also superposed with corresponding AlphaFold2 predicted native structures (Table 2). The RMSD results support the accuracy of AlphaFold2’s predictions, as the predicted native structures are in line with the experimentally determined structures.

**Table 2:** RMSD between native glutamate transporters, their water-soluble QTY variants, and cryo-EM/crystal structures.

Name	PDB ID	RMSD^AF2_Native/Experimental^	RMSD^AF2_QTY/Experimental^
EAA1	5LLM	1.476Å	1.729Å
EAA3	6X2Z	2.126Å	2.456Å

Residue mean-square distance (RMSD) in Å,— = not applicable. All RMSD values are below 3Å and show good superposition between structures.

**Fig 3:**
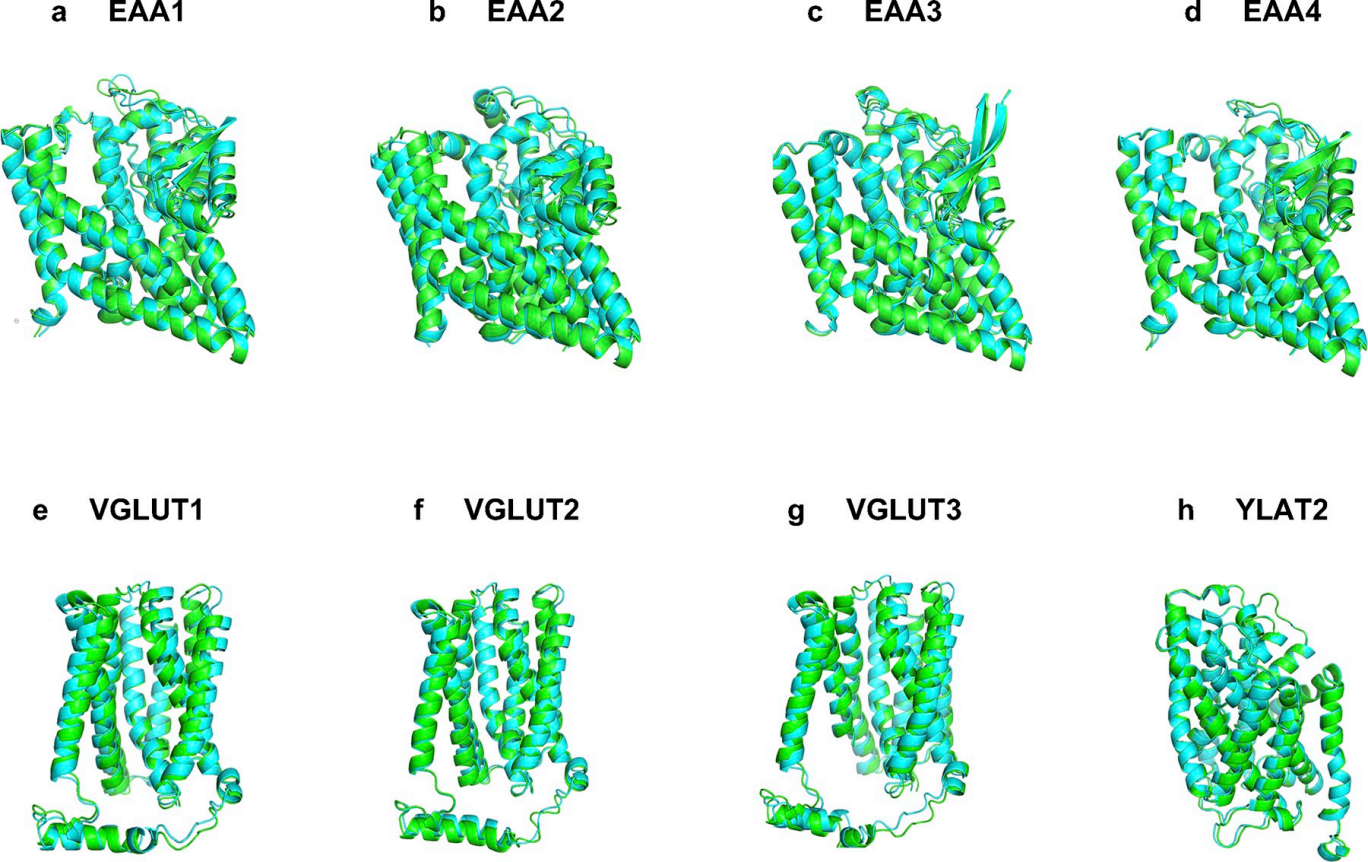
Superposed 8 native glutamate transporters and their QTY variants that were predicted by AlphaFold2. The native structures (green) and their water-soluble QTY variants (cyan). For the superposed structures, the RMSD is in Å (). **a,** EAA1 vs EAA1^QTY^ (0.717Å), **b,** EAA2 vs EAA2^QTY^ (0.948Å), **c,** EAA3 vs EAA3^QTY^ (0.905Å), **d,** EAA4 vs EAA4^QTY^ (0.796Å), **e,** VGLUT1 vs VGLUT1^QTY^ (1.604Å), **f,** VGLUT2 vs VGLUT2^QTY^ (0.971Å), **g,** VGLUT3 vs VGLUT3^QTY^ (1.422Å), **h,** YLAT2 vs YLAT2^QTY^ (0.528Å). For clarity, N- and C- termini and large loops are deleted. Please see Tables 1 and 2.

Many glutamate transporters currently do not have experimentally determined structures, as in the case of numerous other transmembrane proteins. We obtained the structures of six native transporters (EAA2, EAA4, VGLUT1, VGLUT2, VGLUT3, and YLAT2) using AlphaFold2 predictions. Alongside predicted structures of these transporters, AlphaFold2 predicted native EAA1 and EAA3 were also compared with their predicted QTY variants (Table 1 and Fig 3). Despite differences in amino acid composition and chemical characteristics, the structural similarity between the native and QTY variants was high as demonstrated by the root mean square deviation (RMSD). The RMSD values were: EAA1 vs EAA1^QTY^ (0.717Å), EAA2 vs EAA2^QTY^ (0.948Å), EAA3 vs EAA3^QTY^ (0.905Å), EAA4 vs EAA4^QTY^ (0.796Å), VGLUT1 vs VGLUT1^QTY^ (1.604Å), VGLUT2 vs VGLUT2^QTY^ (0.971Å), VGLUT3 vs VGLUT3^QTY^ (1.422Å), YLAT2 vs YLAT2^QTY^ (0.528Å). The native glutamate transporters have four known conformational states, which are classified by the scaffold domain (inward, outward), and the accessibility of the aspartate binding site (open or occluded) namely inward-open, inward-occluded, outward-open, and outward-occluded [49]. The experimental-structures used in this study were outward structures for EAA1 and EAA3 [23, 33]. Meanwhile all AlphaFold2 predicted native and QTY-variant structures also corresponded to the outward-facing structural conformations, meaning the protein core located relatively outward to the rest of the protein (Fig 2). These close alignments reinforce the similarity between the native and water-soluble QTY variants, regardless of hydrophobicity and hydrophilicity (Tables 1 and 2, Figs 2 and 3).

### Analysis of the hydrophobic surface of native transporters and the water-soluble QTY variants

Nature has evolved three types of chemically distinct alpha-helices [50–52]. These are 1) Type I: the hydrophilic alpha-helix, composed mostly of polar amino acids D, E, N, Q, K, R, S, T, and Y [50], as found in water-soluble enzymes and circulating proteins; 2) Type II: the hydrophobic alpha-helix which contains mostly hydrophobic amino acids L, I, V, F, M, P and A [50], present in transmembrane proteins including G protein-coupled receptors, ion channels, the glutamate transporters and transmembrane helices in photosynthesis systems; and (3) Type III: amphiphilic alpha-helices, containing both hydrophobic and hydrophilic amino acid residues. These three types of chemically distinct alpha-helices have similar structures, regardless of their hydrophobicity or hydrophilicity, that is the molecular basis of the QTY code [9].

**Fig 4:**
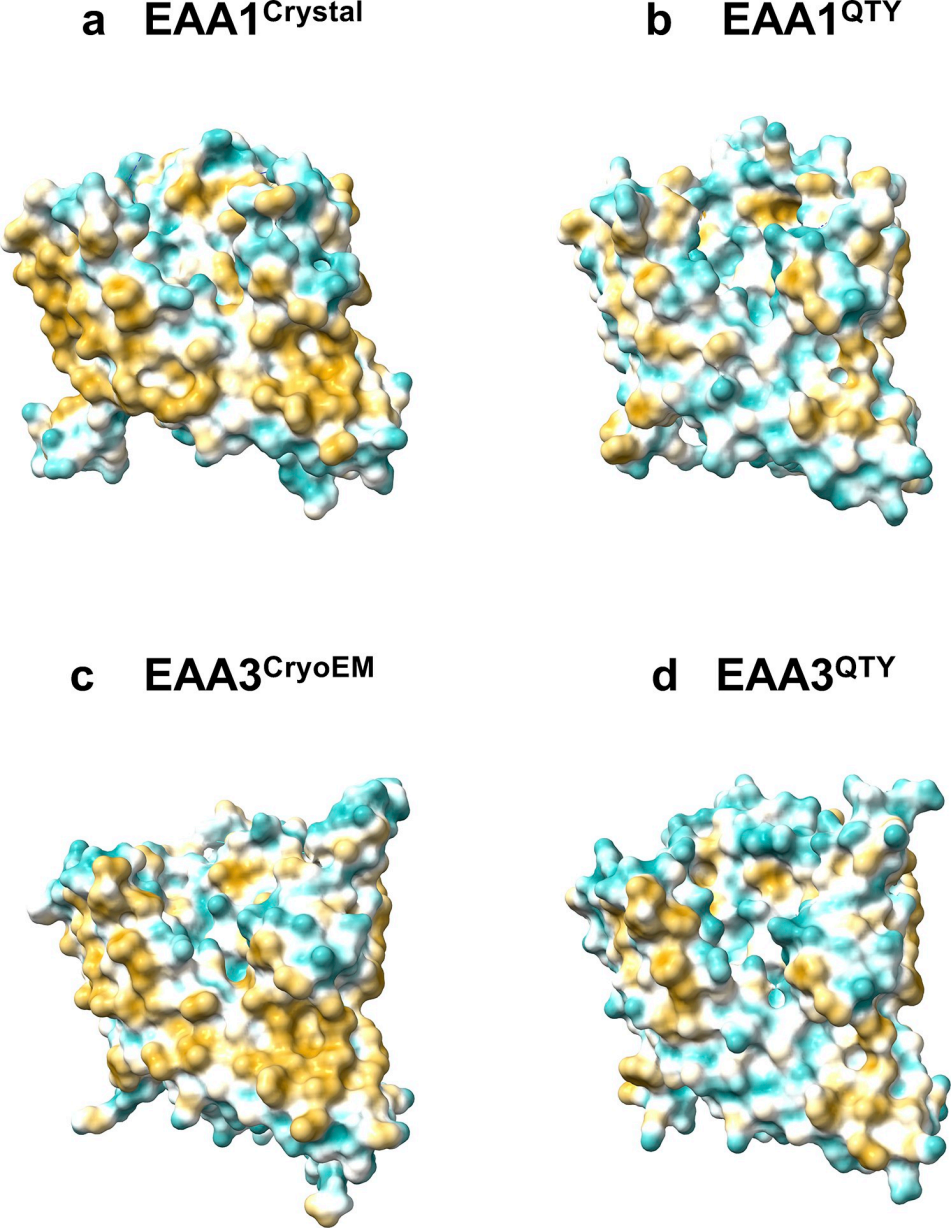
Hydrophobic surface of crystal and cryo-EM structures of two native glutamate transporters and the designed QTY variants. After Q, T, and Y replacement of the hydrophobic residues L, I, V, F, the surfaces were more hydrophilic. The hydrophobic surface (brownish) of the native transporters became more cyan color indicating the hydrophobic surface is largely reduced on the transmembrane helices for the QTY variants: **a** EAA1^Crystal^ vs **b** EAA1^QTY^, **c** EAA3^CryoEM^ vs **d** EAA3^QTY^. For clarity, N- and C- termini and large loops are deleted.

**Fig 5:**
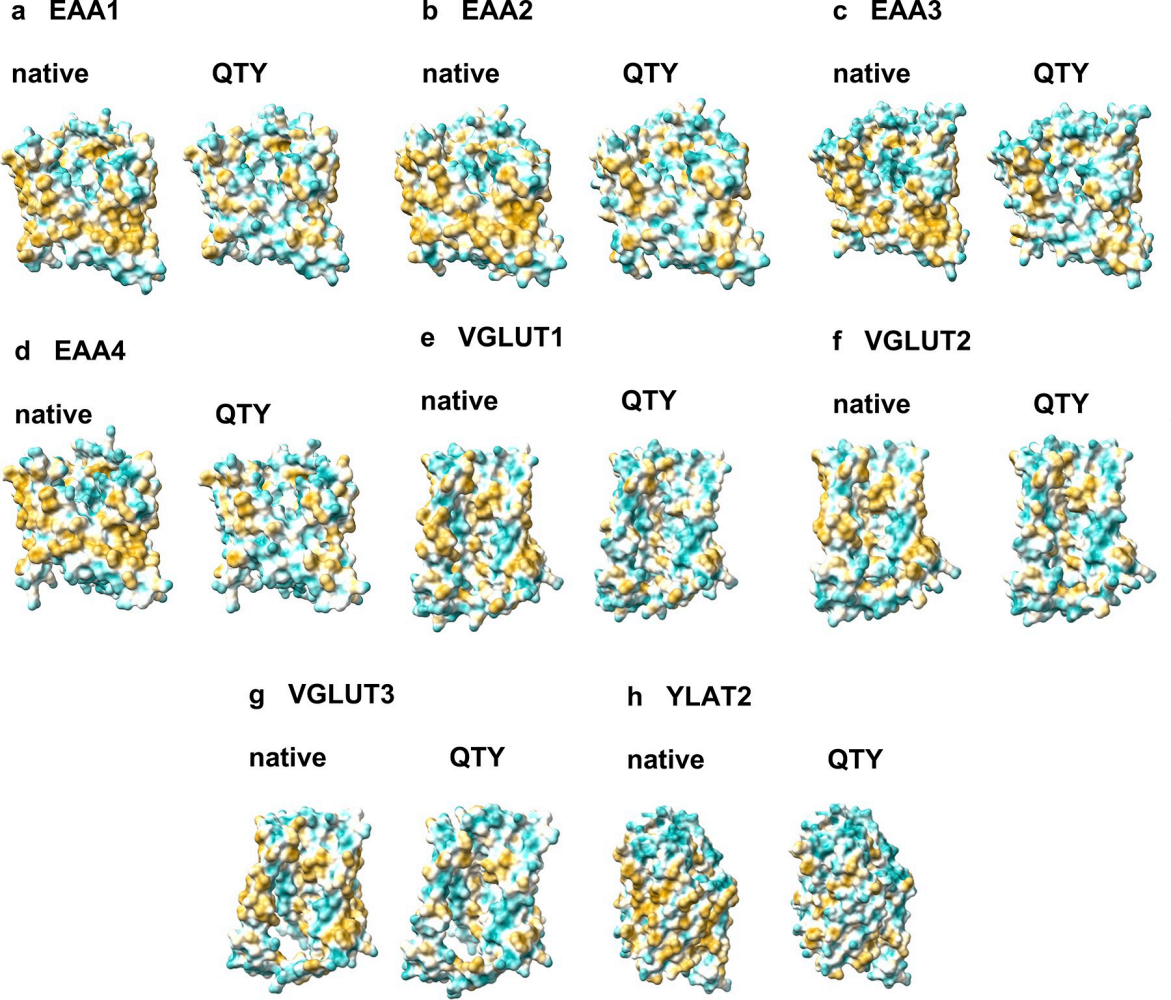
Hydrophobic surfaces of 8 AlphaFold2 predicted native glutamate transporters and their designed QTY variants. After Q, T, and Y replacement of the hydrophobic residues L, I, V, F, the surfaces were more hydrophilic. The hydrophobic surface (brownish) of the native transporters became more cyan color indicating the hydrophobic surface is largely reduced on the transmembrane helices for the QTY variants: **a** EAA1 vs EAA1^QTY^, **b** EAA2 vs EAA2^QTY^, **c** EAA3 vs EAA3^QTY^, **d** EAA4 vs EAA4^QTY^, **e** VGLUT1 vs VGLUT1^QTY^, **f** VGLUT2 vs VGLUT2^QTY^, **g** VGLUT3 vs VGLUT3^QTY^, **h** YLAT2 vs YLAT2^QTY^. For clarity, N- and C- termini and large loops are deleted.

The native structures of glutamate transporters have a high hydrophobicity content, particularly in their transmembrane alpha-helical segments, causing them to be insoluble in water and needing the use of surfactants for isolation [8]. Without these surfactants, the transporters tend to aggregate and form precipitation, leading to a loss of biological function [8]. By replacing the hydrophobic amino acids L, I, V, and F with hydrophilic ones (Q, T, Y), the hydrophobic surfaces were significantly reduced (Figs 4 and 5), this change in hydrophobicity does not disrupt the alpha-helix structure, which was previously unexpected before the systematic experiments were carried out in our recent publications. The experimental evidence that QTY transformation from hydrophobic to hydrophilic transporters retains structural stability and ligand-binding function has been demonstrated in previous studies [9–13]. The QTY code approach is a valuable tool for studying transmembrane proteins, including glutamate transporters. The water-soluble variants of glutamate transporters may not only find potential applications in the design for diagnostic medicine but also in generating monoclonal antibodies and other therapeutics.

### Analysis of genetic variants containing natural mutations of the QTY code

After the improvements in genomics and variant discovery, through the integration of vast data obtained from exome and genome sequencing, the genetic variant analysis found many applications in medical science [53]. This variant analysis may also become a major tool for protein engineering since it provides valuable information on protein variants and their functional effects [54]. Our study analyzed the natural mutations of glutamate transporters and revealed a QTY code that arose from natural processes.

**Fig 6:**
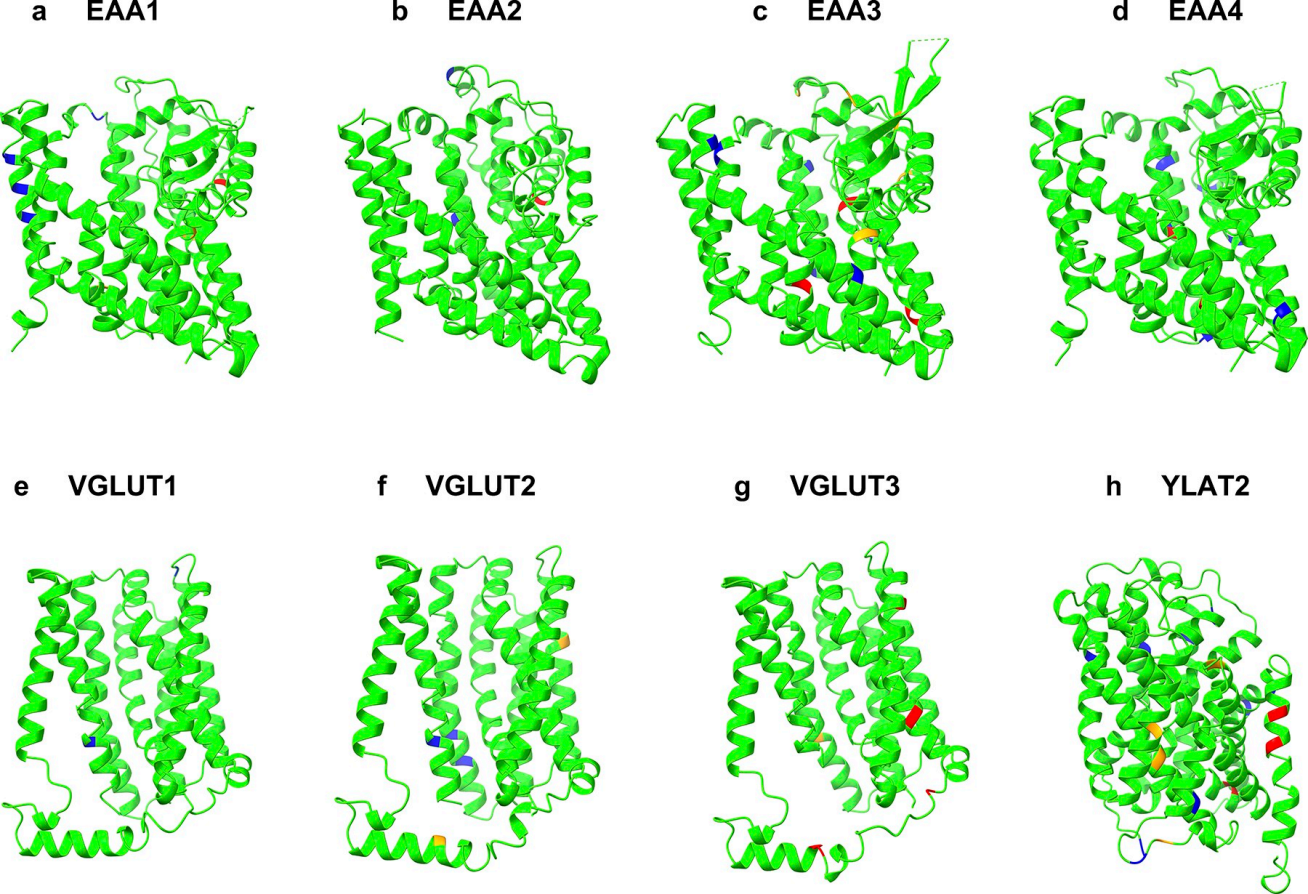
Natural mutations of QTY-code. The native structures (green) and predicted effects of QTY and reverse-QTY mutations are shown as colored residues. Blue = benign, orange = possibly damaging with low confidence, red = damaging with high confidence. **a** EAA1, **b** EAA2, **c** EAA3, **d** EAA4, **e** VGLUT1, **f** VGLUT2, **g** VGLUT3, **h** YLAT2. For clarity, N- and C termini and large loops are deleted.

We used the gnomAD database [37] of 125,748 exomes and 15,708 genomes to survey missense variations of the 8 glutamate transporters. The variants were filtered as QTY (L->Q, V/I->T, F->Y) and reverse QTY (Q->L, T->V/I, Y->F). A total of 95 variants, as 63 QTY and 32 reverse QTY (rQTY), were identified in the glutamate transporter genes. The variations were all single amino acid changes and located at various positions within the transporter protein. The second base of the codon was the only base found to be mutated in all the variations listed, with a total of 95 mutations (Tables 3 and 4). The variations and their predicted effects were visualized (Fig 6 and S21 Fig in S1 File).

**Table 3:** Natural mutations of L->Q, I->T, F->Y in glutamate transporters (No V->T mutations. A single base mutation on the second position of the codons).

Name	Mutation^1^	2^nd^ base^2^	Location^3^	Structure^4^	Exposure^5^	Conservation Grade^6^	Predicted Effect^7^	Clinical Significance^8^
EAA1	I59T	U->C	TM1	α-helix	Exposed	3	benign	-
	I63T	U->C	TM1	α-helix	Exposed	2	benign	uncertain
	I133T	U->C	TM3	α-helix	Exposed	4	? damaging	-
	I214T	U->C	ECL2	loop	-	1	benign	uncertain (EA6^11^)
	I310T	U->C	ECL3	loop	Exposed	5	benign	-
	I349T	U->C	IM	α-helix	Buried	5	damaging	uncertain
	I397T	U->C	TM7	α-helix	Exposed (F^9^)	8	damaging	-
	I526T	U->C	Intracellular	C-coil	-	5	? damaging	-
EAA2	I325T	U->C	TM6	α-helix	Exposed	2	benign	-
	I504T	U->C	Intracellular	α-helix	-	1	benign	-
	I514T	U->C	Intracellular	α-helix	-	3	benign	uncertain
	I522T	U->C	Intracellular	α-helix	-	2	benign	-
EAA3	I72T	U->C	TM2	α-helix	Exposed	7	benign	-
	I90T	U->C	ICL1	α-helix	Buried	8	damaging	uncertain
	I104T	U->C	TM3	α-helix	Exposed	4	benign	uncertain (DCBXA^12^)
	I127T	U->C	ECL2	loop	Buried	1	? damaging	-
	I271T	U->C	ECL3	α-helix	Buried	6	benign	uncertain (DCBXA^12^)
	I298T	U->C	TM6	α-helix	Exposed	4	benign	uncertain (DCBXA^12^)
	I304T	U->C	TM6	α-helix	Exposed	7	? damaging	-
	I307T	U->C	TM6	α-helix	Exposed	3	benign	uncertain (DCBXA^12^)
	I391T	U->C	ECL4	α-helix	Exposed	4	benign	-
	I397T	U->C	IM	α-helix	Buried	8	? damaging	-
	I481T	U->C	Intracellular	α-helix	-	4	benign	-
	L92Q	U->A	ICL1	α-helix	Exposed	3	damaging	-
	L443Q	U->A	TM8	α-helix	Buried	8	damaging	-
	F50Y	U->A	ECL1	α-helix	Exposed	5	benign	benign (DCBXA^12^)
	F508Y	U->A	Intracellular	C-coil	-	7	damaging	-
EAA4	I141T	U>C	TM3	α-helix	Exposed	5	benign	-
	I152T	U>C	TM3	α-helix	Exposed	4	benign	-
	I282T	U>C	TM4	α-helix	Buried	8	benign	-
	I374T	U>C	IM	α-helix	Exposed	4	benign	-
	F351Y	U>A	TM6	α-helix	Exposed	3	benign	-
VGluT1	I128T	U->C	TM2	α-helix	-	6	benign	-
VGluT2	I8T	U->C	Intracellular	loop	-	-	benign	-
	I41T	U->C	Intracellular	β-strand	-	6	benign	-
	I73T	U->C	TM1	α-helix	-	6	? damaging	-
	I286T	U->C	ICL2	α-helix	Exposed	5	? damaging	-
	I365T	U->C	TM8	α-helix	Exposed	3	benign	-
	I369T	U->C	TM8	α-helix	Exposed	4	benign	-
	I482T	U->C	TM12	α-helix	Exposed (F^9^)	8	? damaging	-
VGluT3	I13T	U->C	Intracellular	α-helix	-	6	benign	-
	I100T	U->C	ECL1	α-helix	-	8	damaging	-
	I141T	U->C	TM2	3/10-helix	-	6	damaging	-
	I291T	U->C	ICL3	α-helix	Exposed	6	damaging	-
	I320T	U->C	TM7	α-helix	Buried	6	damaging	uncertain
	I369T	U->C	TM8	α-helix	Exposed	3	? damaging	uncertain (DFNA25^13^)
	I467T	U->C	TM11	α-helix	Exposed	6	damaging	-
	I523T	U->C	Intracellular	loop	-	4	damaging	-
YLAT2	I82T	U->C	TM2	α-helix	Exposed	5	benign	-
	I115T	U->C	TM3	α-helix	Buried	8	damaging	-
	I174T	U->C	TM4	α-helix	Buried	7	? damaging	-
	I204T	U->C	TM5	α-helix	Exposed	2	benign	-
	I283T	U->C	TM7	α-helix	Exposed	5	benign	-
	I336T	U->C	ICL4	α-helix	Buried	6	? damaging	-
	I359T	U->C	ICL4	β-strand	Exposed (F^9^)	8	? damaging	-
	I361T	U->C	ICL4	loop	Exposed	4	benign	-
	I434T	U->C	TM11	α-helix	Exposed	4	benign	-
	I441T	U->C	TM11	α-helix	Exposed	5	benign	-
	I453T	U->C	TM12	α-helix	Exposed	6	damaging	-
	I457T	U->C	TM12	α-helix	Exposed	7	damaging	benign
	I487T	U->C	Intracellular	α-helix	-	1	benign	-
	F343Y	U->A	ICL4	α-helix	Buried	5	? damaging	-
	F387Y	U->A	TM10	α-helix	Exposed	4	? damaging	-

^1^Protein consequence of the mutation according to HGVS numbering.

^2^The second base of the residue codon for the corresponding mutation.

^3^Topological localizations of the mutations according to glutamate transporter molecular architecture (TM = Transmembrane, ECL = Extracellular loop, IM = Intramembrane, ICL = Intracellular loop). The topological information of the mature protein obtained from Uniprot.

^4^Secondary structure of the corresponding residue, calculated from the determined models of native transporters available in the AlphaFold Database.

^5^Residue exposure according to the NACSES algorithm, predicted by ConSurf server

^6^Evolutionary conservation grade of the residue predicted by ConSurf server; 1 to 9, in order of increasing conservation (1 = Variable, 5 = Average, 9 = Conserved).

^7^Variant effect predicted by Polyphen. Benign = predicted to be benign with high confidence;? damaging = possibly damaging, predicted to be damaging with low confidence; damaging = probably damaging: predicted to be damaging with high confidence.

^8^Based on ClinVar’s January 21, 2023 release.

^9^A functional residue (exposed and highly conserved) predicted by ConSurf Server.

^10^A structural residue (buried and highly conserved) predicted by ConSurf Server.

^11^EA6 = Episodic ataxia type 6

^12^DCBXA =  Dicarboxylic aminoaciduria

^13^DFNA25 = Autosomal dominant nonsyndromic hearing loss 25

**Table 4:** Natural mutations of Q->L, T->I, Y->F in glutamate transporters (No T->V mutations. A single base mutation on the second position of the codons).

Name	Mutation^1^	2^nd^ base^2^	Location^3^	Structure^4^	Exposure^5^	Conservation Grade^6^	Predicted Effect^7^	Clinical Significance^8^
EAA1	T2I	C->U	Intracellular	N-coil	-	-	benign	-
	T67I	C->U	TM1	α-helix	Exposed	3	benign	-
	T225I	C->U	ECL2	β-strand	-	1	benign	-
	T428I	C->U	IM	α-helix	Exposed	7	damaging	-
EAA2	T433I	C->U	IM	α-helix	Buried (F^9^)	9	damaging	-
	Q152L	A->U	ECL2	α-helix	-	3	benign	-
EAA3	T133I	C->U	ECL2	loop	Exposed	3	? damaging	-
	T164I	C->U	ECL2	β-strand	Buried (S^10^)	9	? damaging	-
	T197I	C->U	ECL2	loop	-	4	? damaging	-
	T340I	C->U	IM	α-helix	Buried (S^10^)	9	damaging	-
	T364I	C->U	TM7	α-helix	Buried (S^10^)	9	? damaging	-
	T370I	C->U	TM7	α-helix	Buried (S^10^)	9	damaging	-
	Y285F	A->U	ECL3	α-helix	Buried	8	benign	-
EAA4	T343I	C>U	ECL3	α-helix	Buried	6	benign	-
	T366I	C>U	ICL3	α-helix	Buried	8	benign	-
	T412I	C>U	TM7	α-helix	Buried	8	damaging	-
	T507I	C>U	TM8	α-helix	Buried	5	damaging	-
	Q27L	A>U	Intracellular	α-helix	-	5	benign	-
	Q549L	A>U	Intracellular	α-helix	-	6	? damaging	-
VGluT1	T96I	C->U	ECL1	β-strand	-	3	benign	-
	T209I	C->U	TM5	α-helix	Exposed	4	benign	-
	T464I	C->U	ECL5	loop	Exposed	7	benign	-
VGluT2	T164I	C->U	TM3	α-helix	-	4	benign	-
	T217I	C->U	TM5	α-helix	Exposed	5	benign	-
VGluT3	T40I	C->U	Intracellular	loop	-	4	benign	-
	T119I	C->U	ECL1	loop	-	1	benign	uncertain
	T305I	C->U	ICL3	loop	Buried	7	damaging	-
	T384I	C->U	ICL4	loop	Exposed	7	damaging	-
	T551I	C->U	Intracellular	loop	-	5	damaging	-
YLAT2	T10I	C->U	Intracellular	N-coil	-	-	benign	-
	T74I	C->U	ECL1	α-helix	Buried	6	? damaging	-
	Q40L	A->U	Intracellular	N-coil	Exposed	1	benign	-

^1^Protein consequence of the mutation according to HGVS numbering.

^2^The second base of the residue codon for the corresponding mutation.

^3^Topological localizations of the mutations according to transporter molecular architecture (TM = Transmembrane, ECL = Extracellular loop, IM = Intramembrane, ICL = Intracellular loop). The topological information of the mature protein obtained from Uniprot.

^4^Secondary structure of the corresponding residue, calculated from the determined models of native transporters available in the AlphaFold Database.

^5^Residue exposure according to the NACSES algorithm, predicted by ConSurf server

^6^Evolutionary conservation grade of the residue predicted by ConSurf server; 1 to 9, in order of increasing conservation (1 = Variable, 5 = Average, 9 = Conserved).

^7^Variant effect predicted by Polyphen. Benign = predicted to be benign with high confidence;? damaging = possibly damaging, predicted to be damaging with low confidence; damaging = probably damaging: predicted to be damaging with high confidence.

^8^Based on ClinVar’s January 21, 2023 release.

^9^A functional residue (exposed and highly conserved) predicted by ConSurf Server.

^10^A structural residue (buried and highly conserved) predicted by ConSurf Server.

The variations were distributed across different domains of the transporter protein. Topological localization of the QTY mutations according to glutamate transporter molecular architecture revealed that 34 out of 63 (54.0%) of the variations were located in the transmembrane (TM) regions. This finding can be attributed to the presence of polar L, I, and F amino acids within the TM helices. For the predicted effects of these variations, predictions from Polyphen-2 [38] defined 19 of those as benign (19/34 = 55.9%), 7 as “possibly” damaging with low confidence (7/34 = 20.6%), and 8 as probably damaging (8/34 = 23.5%).

Twenty-nine of the natural QTY mutations were outside the TM domain, corresponding to ~46.0%. Specifically, three mutations were found in the intramembrane regions, 7 in the extracellular regions, and 19 in the cytoplasmic regions. As a result, 15 of the mutations were predicted to be benign (15/29 = 51.7%), 7 as “possibly” damaging with low confidence (7/29 = 24.1%), and 7 as probably damaging (7/29 = 24.1%). Notably, regardless of their location, more than half of the natural QTY mutations were predicted to be benign (Table 3). Per-residue secondary structure assignment from AlphaFold2 determined models showed that 53 mutations belong to a helical structure, and 29 of those were benign (Table 3).

On the other hand, 32 natural reverse-QTY (Q->L, T->V/I, Y->F) mutations examined in this study were predominantly found outside the TM regions (24/32 = 75%). In detail, three of the rQTY mutations were found in the intramembrane regions, 11 in the extracellular regions, and 10 in the cytoplasmic regions. Outside the TM regions, 13 mutations were predicted to be benign (13/24 = 54.2%), 5 as “possibly” damaging with low confidence (5/24 = 20.8%), and 6 as probably damaging (6/24 = 25.0%). Regardless of their location, 17 out of 32 (53.1%) of the reverse QTY mutations were predicted to be benign. Secondary structure assignment data showed that 18 mutations belong to a helical structure, and 9 of those were benign (Table 4).

The ClinVar archives [22] demonstrated the clinical effects of 13 natural QTY or rQTY substitutions (Tables 3 and 4). Two of the variants reported in the ClinVar database were benign (VCV000367038.7 and VCV000777038.3) and a total of 11 variants were associated with uncertain significance in three different conditions: episodic ataxia type 6 (VCV000906384.2), dicarboxylic aminoaciduria (VCV001701474.3, VCV000994967.1, VCV001373953.2, VCV000212195.5, VCV000913887.2, VCV000367050.3, VCV000367048.3, VCV000367041.3, VCV000883186.2), and autosomal dominant nonsyndromic hearing loss 25 (VCV001304165.2).

### Natural mutations of L->Q, I->T, F->Y and Q->L, T->I, Y->F in glutamate transporters

The Genetic code’s second position determines the chemical nature of amino acids [55, 56]. For example, i) amino acids with U at the second position are hydrophobic (Phe, Leu, Ile, Val, and Met); ii) amino acids with C at the second position are less hydrophobic (Pro and Ala), or with a hydroxyl -OH group (Ser and Thr); iii) amino acids with A at the second position are hydrophilic and water soluble (Asp, Glu, Asn, Glu, Lys, His and Tyr), and 2 stop codons Ochre (UAA) and Amber (UAG); iv) amino acids (Arg and Ser) with G at the second position are water soluble, Cys is partially water-soluble and Gly is achiral and has an H as the side chain [55, 56]. The stop codon is UGA. In general, pyrimidine U and C at the second position confer hydrophobicity; in contrast, purine A and G at the second position confer hydrophilicity (S1 Fig in S1 File).

In the glutamate transporters, there are many natural mutations of L->Q, I->T, F->Y and Q->L, T->I, Y->F. These mutations result from a single nucleotide change, all occur in the second position of the genetic code, including transition mutation, i.e., purine to purine (A->G, G->A) and pyrimidine to pyrimidine (C->U, or U->C); or transversion mutation (U->A, U->G, C->A, C->G, A->U, A->C, G->U, G->C).

In the case of L->Q, I->T, and F->Y mutations. For example, i) in L (leucine), two codons are CUA and CUG, and in Q (glutamine), two codons are CAA and CAG; in these cases, the second position of U is mutated to A, which is a transversion mutation. ii) In I (isoleucine), three codons are AUU, AUC, and AUA, in T (threonine), four codons are ACU, ACC, ACA, and ACG; in these cases, the second position of U is mutated to C which is a transition mutation. iii) In F (phenylalanine), two codons are UUU and UUC, in Y (tyrosine), two codons are UAU and UAC, and the second position of U is mutated to A which is a transversion mutation.

Likewise, in the mutations of Q->L, T->I, Y->F, it is the change of Q, T, Y to L, I, F. Namely, i) in Q (glutamine), two codons are CAA and CAG, when the codons are mutated to CUA and CUG, they changed to L (leucine). ii) Four codons of T (threonine) are ACU, ACC, ACA, and ACG, when they are mutated to AUU, AUC, and AUA which is the transition mutation, they changed T to I (isoleucine). iii) Following the same logic, two codons of Y (tyrosine) are UAU and UAC, when they are mutated to UUU and UUC which is a transversion mutation, the Y is changed to F.

No V->T, nor T-> V mutations in the transporters are observed (Tables 3 and 4). This is because such changes require at least 2 nucleotide changes. The four valine (V) codons are GUU, GUC, GUA, and GUG, and the four threonine (T) codons are ACU, ACC, ACA, and ACG. In this study, we only focused on the QTY relevant mutations and did not systematically examine other mutations since it is beyond the scope of this study.

### QTY and rQTY mutation libraries

Mutation libraries are an essential tool for modern genetic and medical analysis. By collectively analyzing a diverse set of genetic variants, mutation libraries provide researchers and medical doctors with the means to investigate variants for desired traits, such as stability or phenotypical effects. These libraries are typically constructed through a process of in vivo and in vitro mutagenesis [57]. In contrast, hereby we present the comprehensive genetic analysis using solely computational methods, which may be notably faster and less costly than conventional mutagenesis.

For the analysis of the amino acid residues which naturally occurred QTY and reverse QTY (rQTY) variations were submitted by large-scale sequencing projects, we built mutation libraries by calculating the effects of all 19 amino acid substitutions possible to occur at the residue, except the wild amino acid. In total, more than 1,800 potential variations and their impacts on the native protein were predicted. The Polyphen-2 algorithm considers hydrophobic potentials when predicting the effects of amino acid substitutions on protein function and structure [38]. As a result, substitutions to the polar amino acids leading to soluble variants may be expected to have a higher predicted score since they are unlikely to be found in the proteins on the cell membrane. However, these substitutions may not necessarily change the overall structure of the protein, as the alignment results suggest. Accordingly, to further investigate the natural QTY variations, we compared the effects of naturally occurred substitutions of L->Q, I->T, and F->Y, which are polar, to substitutions involving other polar amino acids including L to D, E, R, K, H, N, S, T, Y; I to D, E, R, K, H, N, S, Q, Y; and F to D, E, R, K, H, N, S, T, Q.

The PolyPhen-2 calculations showed that the natural QTY code variations are notably less damaging compared to the average of other polar amino acid changes. For the residue where the natural QTY code variations occurred, the average pph2_prob score (represents the probability of a substitution being damaging, ranges from 0.0 to 1.0) for other polar amino acid substitutions was 0.725, whereas for the QTY code substitutions, it was 0.588. The natural QTY substitutions also showed a lower impact compared to the average of all 19 amino acids (0.648), regardless of their polarity. This is perhaps due to the similar molecular structures of L, I/V, F with Q, T, Y, respectively at particular position, thus these mutations have less change for the molecular structures.

For analyzing reverse QTY (rQTY) mutations, we compared the effects of naturally occurring substitutions of Q->L, T->I, and Y->F, to substitutions involving other nonpolar amino acids (A, C, G, I, L, M, F, P, W, V). The PolyPhen-2 calculations again showed that the rQTY variations are significantly less damaging compared to the average of other nonpolar amino acid changes. For the residue where the rQTY code variations occurred, the average pph2_prob score for other nonpolar amino acid substitutions was 0.562, and for the rQTY substitutions, it was just 0.339. Moreover, the rQTY substitutions also showed a prominently lower impact compared to the average of all 19 amino acids (0.541), regardless of their polarity. 3D plots were drawn to visualize the predicted effect of 19 possible variations of the residue of which natural QTY and rQTY substitutions were submitted by sequencing projects (S8 and S9 Figs in S1 File). These findings can also be reasoned with the explanation described above.

### Evolutionary conservation studies and analysis of sensitive domains

Glutamate transporters play a vital role in the central nervous system (CNS) by removing excess glutamate from the synapse, involving fundamental mechanisms [1, 2]. Furthermore, the structural mechanism of amino acid symport that is prominent in the glutamate transporters, is evolutionarily conserved in diverse species from archaea to humans [23]. Evolutionary conservation analysis of the amino acid sequence of 8 native glutamate transporters showed that many residues are in fact highly conserved, indicating their functional and evolutionary significance (S20 Fig in S1 File). The number of residues that have more than average conservation grade was calculated as follows: ~68.3% for EAA1 (285/417), ~69.5% for EAA2 (287/413), ~70.3% for EAA3 (298/424), ~69.5% for EAA4 (290/417), ~61.2% for VGLUT1 (180/294), ~62.9% for VGLUT2 (185/294), ~61.0% for VGLUT3 (175/287), ~62.6% for YLAT2 (274/438).

Interestingly, transmembrane (TM) regions of glutamate transporters were found to be more conserved compared to the motifs in the N- and C- termini (S12-S19 Figs in S1 File). This conservation may be attributed to the crucial role played by TM regions in maintaining the structural integrity of these proteins. In support of this, mutation visualization of the whole transporter sequence also showed that the residues at the TM domains are more sensitive to amino acid substitutions compared to the N-termini and C-termini (S10 and S11 Figs in S1 File). As expected from the evolutionary profiling, EAATs were also found to be more sensitive to mutations than VGLUTs (S10 and S11 Figs in S1 File, respectively).

Despite many residues of glutamate transporters being evolutionarily conserved, the Q, T, Y mutations did not affect the overall predicted structure, and AlphaFold 2 predicted QTY variants superposed well with native structures. To further analyze the phenotypical effects of QTY code on the TM regions, alongside the natural variant analysis derived from genomic databases, we also built mutation libraries for all L, I, and F amino acids in the TM region of the EAA1 (total 97), regardless of their occurrence in the population or nature. The results showed that the TM regions are indeed sensitive to changes, confirming the evolutionary data and mutation visualizations of the entire sequence. The impact of the substitutions varied (S3-S7 Figs in S1 File). For instance, the substitution of L (leucine) with other nonpolar amino acids such as I (isoleucine) is predicted to have less impact on EAA1 function than substitution with polar amino acids (S3 Fig in S1 File). Substitution of I at certain positions in TM segments had minor impacts on protein function (S4 Fig in S1 File). Substitutions from F also had similar pattern with those from I and L, indicating effects of polarity on the amino acid substitutability (S5 Fig in S1 File). One possible explanation for this observation could be the structural similarity between I and V (as well as L), as their branched side chains allow for similar interactions. Such findings suggest that substituting certain amino acid residues that share similar structures may not significantly alter protein structure or function, aligning with the primary hypothesis of the QTY code [9]. Regarding the primary focus of this study, the L->Q, I->T, F->Y substitutions (QTY code) had a slightly lower impact on function and structure (~0.819), compared to the average of the 19 amino acids (~0.825), and were notably less damaging than the average of other polar amino acids (~0.896).

### Possible implications and future directions of the study

Our study provides insights into the influence of amino acid substitutions in the transmembrane (TM) region of the glutamate transporters, offering approaches to design diagnostics tools, and generate therapeutics monoclonal antibodies. Even if the TM domains are sensitive to substitutions and under strong evolutionary conservation, our findings suggest that it may be possible to create soluble variants of these domains that do not perhaps alter the overall structure of the transporters. Membrane localization also regulates the dynamics of native glutamate transporters, hence contributing to the transport process [23, 33]. In the case of designed soluble variants, their potential additional functions that differ from wild type proteins (such as solubility) may also generate valuable research outcomes. Performing Molecular Dynamics simulations can facilitate the study of functional properties that result from differences in water accessibility [58, 59]. While it may not be easy to explain their functional dynamics and behavior in soluble environments, and well beyond the scope of structural informatics analysis, our study utilizing the phenotypical profiling shed light upon the roles of TM segments and their bilayer localization in transport function. Even if the QTY variants cannot perform some functions that are specific to wild type protein’s membranous localization, taking into account that such stable soluble variants share substantial structural composition with their transmembrane counterparts, makes them strong tools for both functional studies and drug design. Such outcome results from targeting soluble proteins is easier than those involving membrane proteins [8]. Having similar structural conformations as its native counterparts, QTY variants could potentially be utilized with the existing pharmaceutical discovery strategies [33]. Furthermore, this structural alignment with native transporters suggests that the QTY variants can also provide valuable tools to produce antibodies for effectively managing various disorders, especially when considering the already existing studies on roles of anti-EAA2 autoantibodies in disease etiologies [60]. This characteristic is therefore specific to soluble QTY variants and could not be achieved with native membrane proteins. Molecular Dynamics simulations could be further used to explain the mutagenesis induced dynamics of the variants and specific amino acid substitutions [61, 62]. Since our study focused on the theoretical aspects, experimental studies involving QTY variants are likely to be beneficial. We suggest further experimental research to consider these specific functional differences and additional applications resulting from the unstudied dynamics of water-soluble TM-like segments, at the same time we further emphasize the similarities of our suggested QTY-code with the reverse QTY-code.

## Conclusion

Our study moreover considers evolutionary aspects of the QTY-code design strategy. Such analysis is especially useful for genetic variant analysis since the phenotypical or functional differences cannot always be causally linked with genetic variants, which may therefore become a major limitation of protein design strategies using genetic variant analysis [63, 64]. Through our analysis of genetic variations submitted by large-scale sequencing studies, we uncovered the potential to trace less harmful systematic variations for effective protein design.

Our findings suggest that variant analysis and evolutionary profiling, combined with structural informatics studies, are promising research tools for designing proteins with specific properties, such as water solubility. Accordingly, our data revealed that the QTY code did not alter the overall structure of the 8 glutamate transporters. Moreover, the QTY code had a notably lesser impact on the phenotypical characteristics of the proteins under investigation, as compared to the average of other polar amino acid substitutions.

Our structural bioinformatics studies not only provided insight into the differences between the hydrophobic helices and hydrophilic helices in the glutamate transporters, but they are also expected to stimulate further study of other water-soluble transmembrane proteins.

## Supporting information

S1 File Contains all the supporting S1-S22 Figs with captions in a separate file. (DOCX)
