# Supplementary material for: Structural bioinformatics studies of glutamate transporters and their AlphaFold2 predicted water-soluble QTY variants and uncovering the natural mutations of L->Q, I->T, F->Y and Q->L, T->I and Y->F
Source: PLoS One. 2024 Apr 10;19(4):e0289644. doi: 10.1371/journal.pone.0289644 (PMC11006163; doi:10.1371/journal.pone.0289644)
Supplement: S1 File — (DOCX) [file pone.0289644.s001.docx]

Supporting Information

Structural bioinformatics studies of glutamate transporters and their AlphaFold2 predicted water-soluble QTY variants and uncovering the natural mutations of L->Q, I->T, F->Y and Q->L, T->I and Y->F

Alper Karagöl^1,¶^, Taner Karagöl^1,¶^, Eva Smorodina^2^, Shuguang Zhang^3,*^

^1^Istanbul University Istanbul Medical Faculty, Istanbul, Turkey

^2^Laboratory for Computational and Systems Immunology, Department of Immunology, University of Oslo, Oslo University Hospital, Oslo, Norway

^3^Laboratory of Molecular Architecture, Media Lab, Massachusetts Institute of Technology, 77 Massachusetts Avenue, Cambridge, MA, 02139, USA

^¶^These authors contribute equally.

*To whom the correspondence should be addressed.

Email:

Alper Karagöl, [alper.karagol@gmail.com](mailto:alper.karagol@gmail.com) ORCID: [0009-0001-7864-0732](https://orcid.org/0009-0001-7864-0732)

Taner Karagöl, [taner.karagol@gmail.com](mailto:taner.karagol@gmail.com) ORCID: [0009-0005-1011-7661](https://orcid.org/0009-0005-1011-7661)

Eva Smorodina, [ribes.ev@gmail.com](mailto:ribes.ev@gmail.com) ORCID: [0000-0002-5457-5163](https://orcid.org/0000-0002-5457-5163)

Shuguang Zhang, [Shuguang@MIT.EDU](mailto:Shuguang@MIT.EDU) ORCID: [0000-0002-3856-3752](https://orcid.org/0000-0002-3856-3752)

**
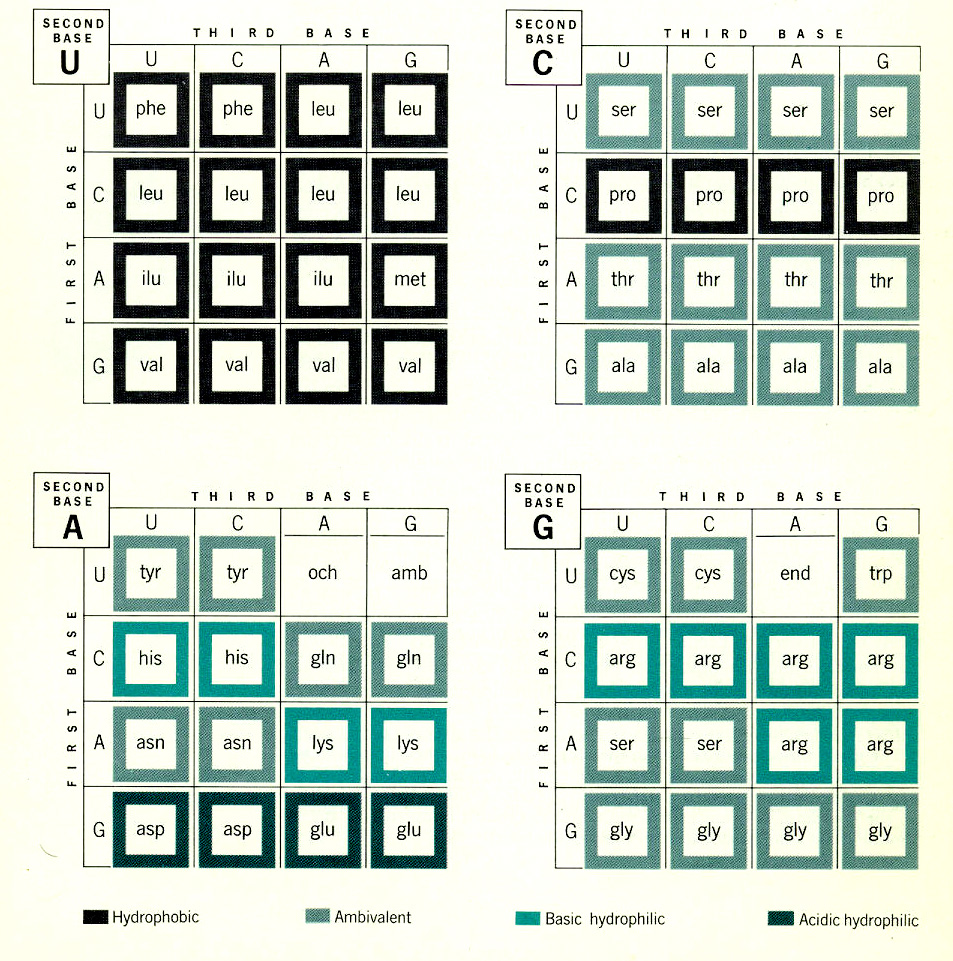
**

**Figure S1. The second position importance of Genetic code.** The Genetic code’s second position determines the chemical nature of amino acids. Their second position is emphasized. For example, ***i***) amino acids with U at the second position are hydrophobic (Phe, Leu, Ile, Val and Met); ***ii***) amino acids with C at the second position are less hydrophobic (Pro and Ala), or with a hydroxyl OH group (Ser and Thr); ***iii***) amino acids with A at the second position are hydrophilic and water soluble (Asp, Glu, Asn, Glu, Lys, His and Tyr), and 2 stop codons Ochre (UAA) and Amber (UAG); ***iv***) amino acids (Arg and Ser) with G at the second position are water soluble, Cys is partially water-soluble and Gly is achiral and has a H as the side chain. The stop codon is UGA. In general, pyrimidine U and C at the second position confer hydrophobicity; in contrast, purine A and G at the second position confer hydrophilicity.


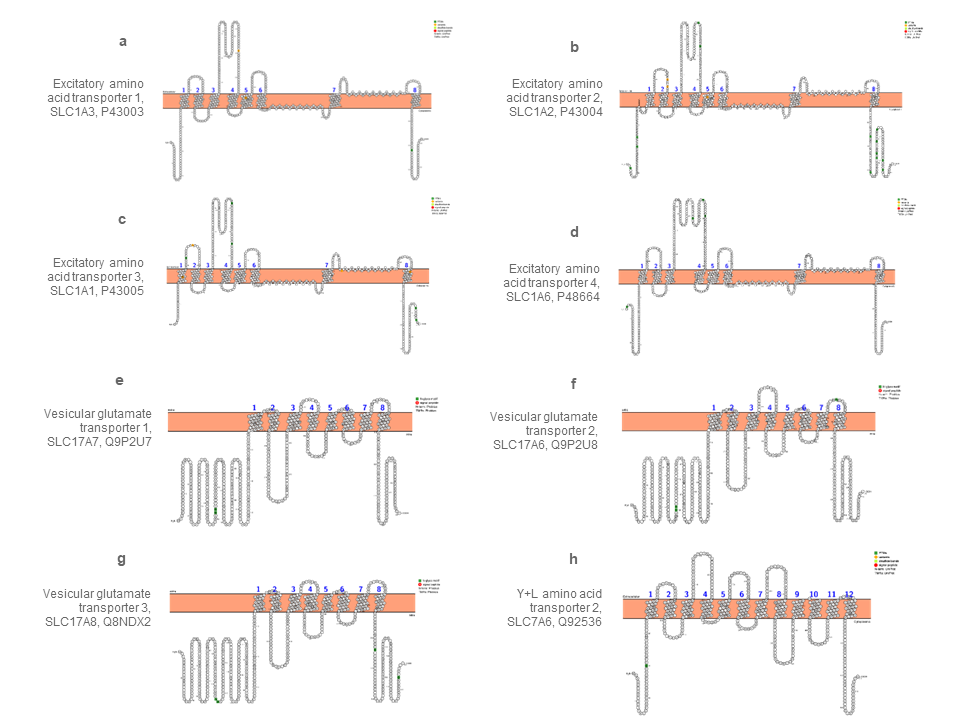


**Figure S2. Membrane topology models of eight native glutamate transporter.** Prediction was performed with the Protter web client, based on the sequence analysis of the corresponding transporter using the UniProt database. EAATs and VGLUTs has an 8-transmembrane (TM) architecture, while the Y+L amino acid transporter-2 (YLAT2) has 12 TM domain. EAATs have a larger extracellular loop between TM3 and TM4, meanwhile VGLUTs have a larger portion of intracellular motifs than those in EAATs and YLAT2.


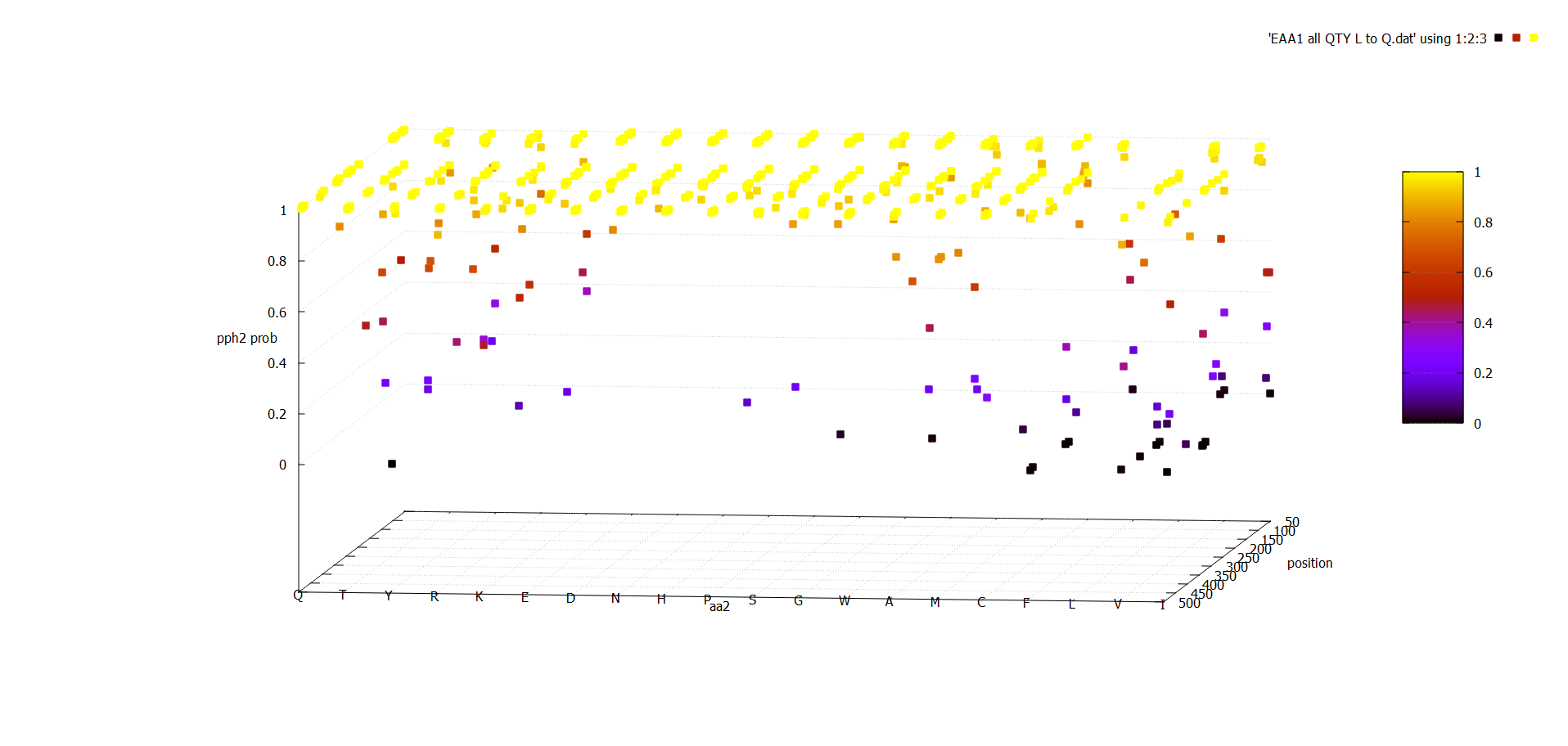


**Figure S3. Predicted effects of variations at the L amino acid residue located in the TM segments of EAA1.** The x-axis indicates the second amino acid that replaced the wild-type (L) amino acid residue, while the y-axis and color scale represent the PolyPhen-2 predicted effect of the substitution, ranging from benign (0.0) to damaging (1.0). The z-axis indicates the position of the substitution within the native protein sequence.


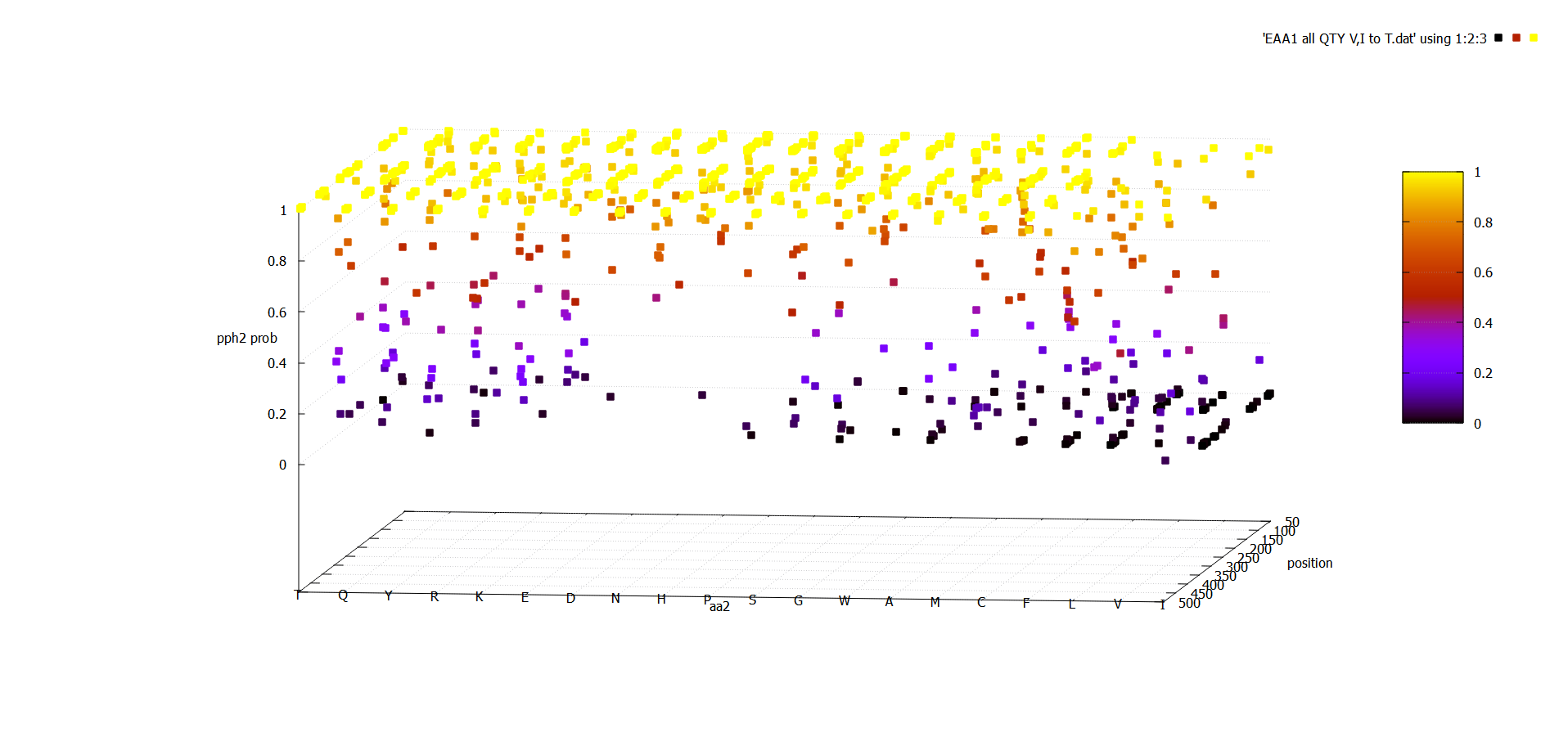


**Figure S4. Predicted effects of variations at the V & I amino acid residue located in the TM segments of EAA1.** The x-axis indicates the second amino acid that replaced the wild-type (V or I) amino acid residue, while the y-axis and color scale represent the PolyPhen-2 predicted effect of the substitution, ranging from benign (0.0) to damaging (1.0). The z-axis indicates the position of the substitution within the native protein sequence.


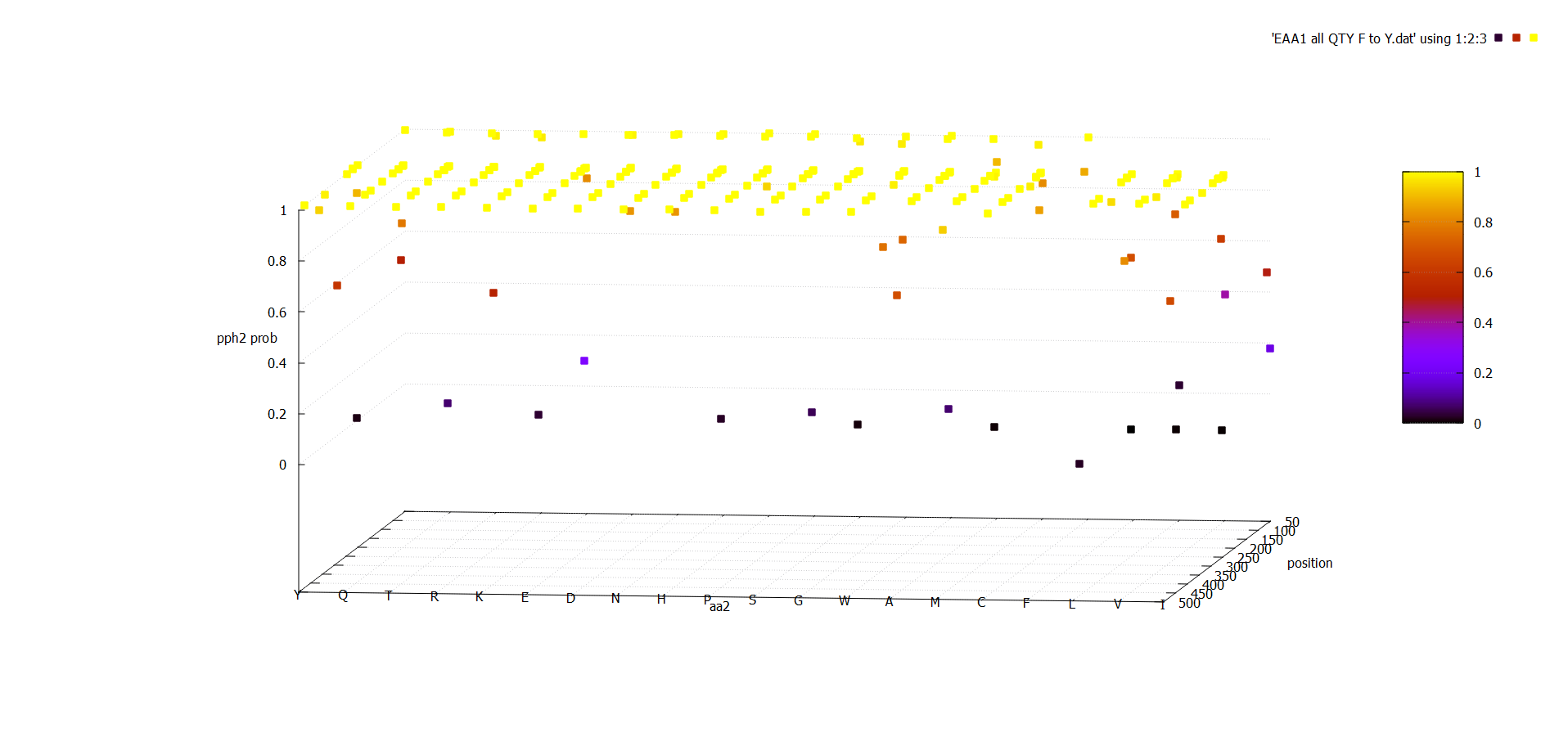


**Figure S5. Predicted effects of variations at the F amino acid residue located in the TM segments of EAA1.** The x-axis indicates the second amino acid that replaced the wild-type (F) amino acid residue, while the y-axis and color scale represent the PolyPhen-2 predicted effect of the substitution, ranging from benign (0.0) to damaging (1.0). The z-axis indicates the position of the substitution within the native protein sequence.


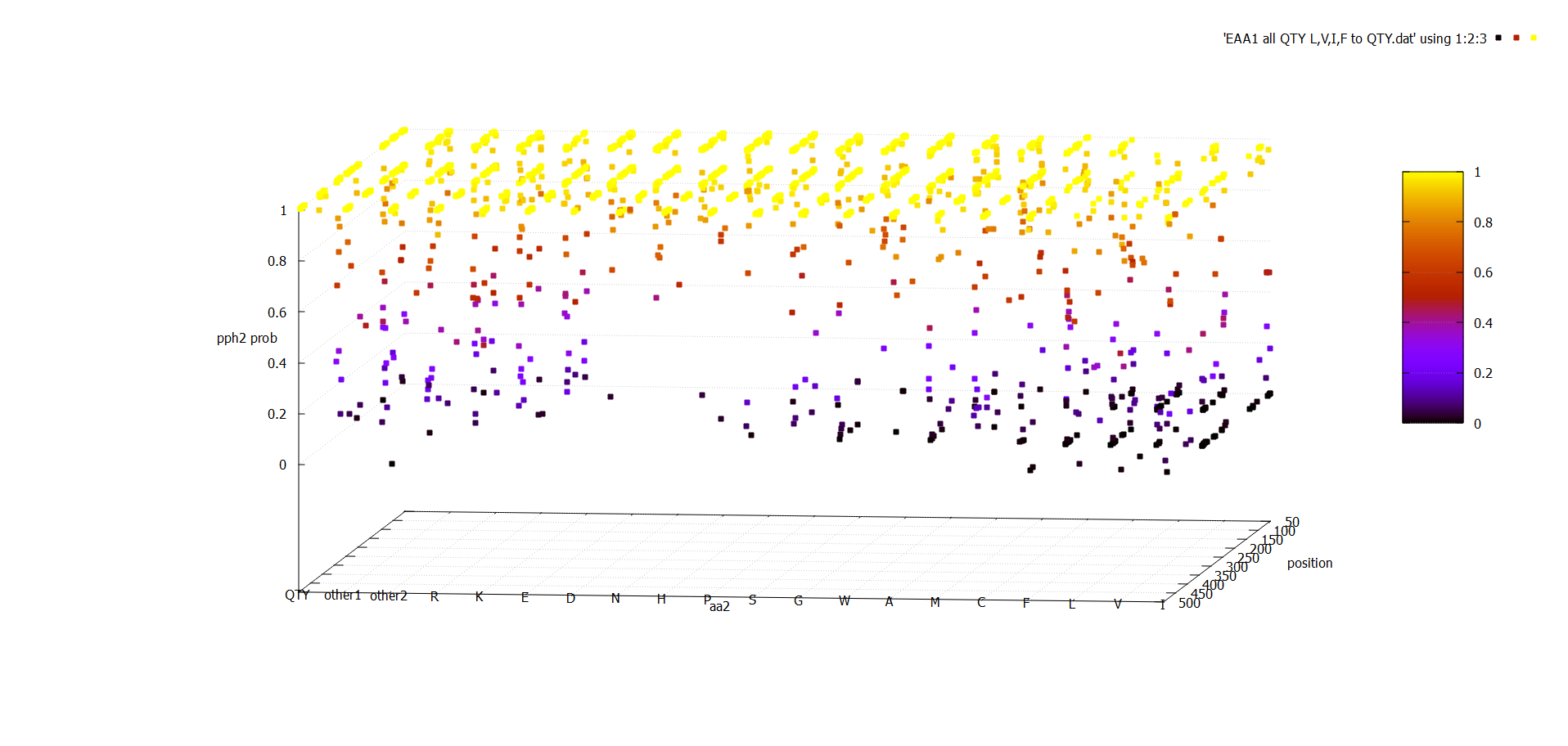


**Figure S6. Predicted effects of the L->Q, I&V->T, F->Y variations in the TM segments of EAA1.** The x-axis indicates the second amino acid that replaced the wild-type (L, I, V and F) amino acid residue, while the y-axis and color scale represent the PolyPhen-2 predicted effect of the substitution, ranging from benign (0.0) to damaging (1.0). The z-axis indicates the position of the substitution within the native protein sequence. QTY is the L->Q, I&V->T,and F->Y substitutions; other1 and other2 are the Q, T, Y amino acid substitutions which is different than the QTY code (I, V, F->Q and L, F->T and L, I, V->Y)


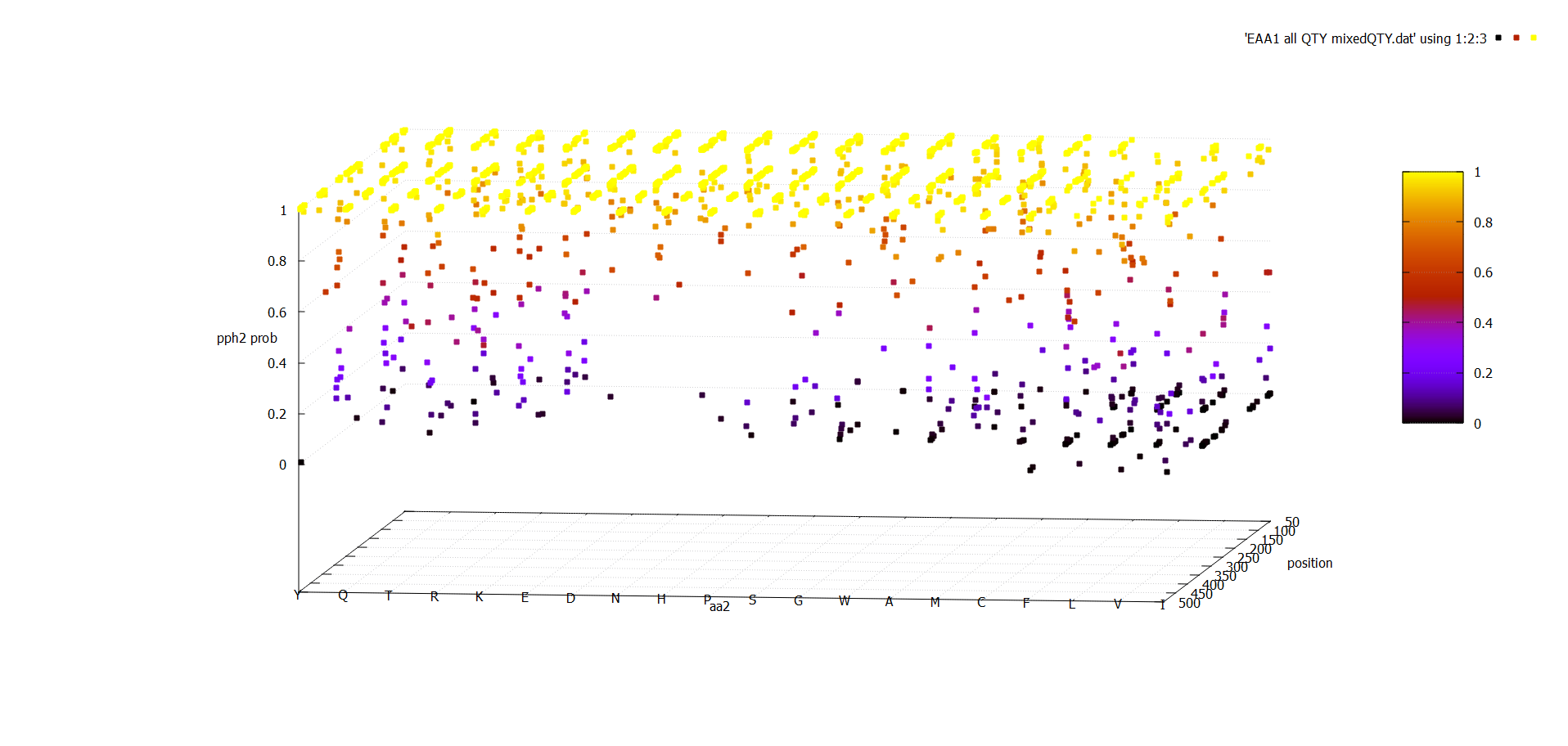


**Figure S7. Predicted effects of the variations at the L, V, I, F amino acid residues in the TM segments of EAA1.** The x-axis indicates the second amino acid that replaced the wild-type (L, I, V and F) amino acid residue, while the y-axis and color scale represent the PolyPhen-2 predicted effect of the substitution, ranging from benign (0.0) to damaging (1.0). The z-axis indicates the position of the substitution within the native protein sequence.


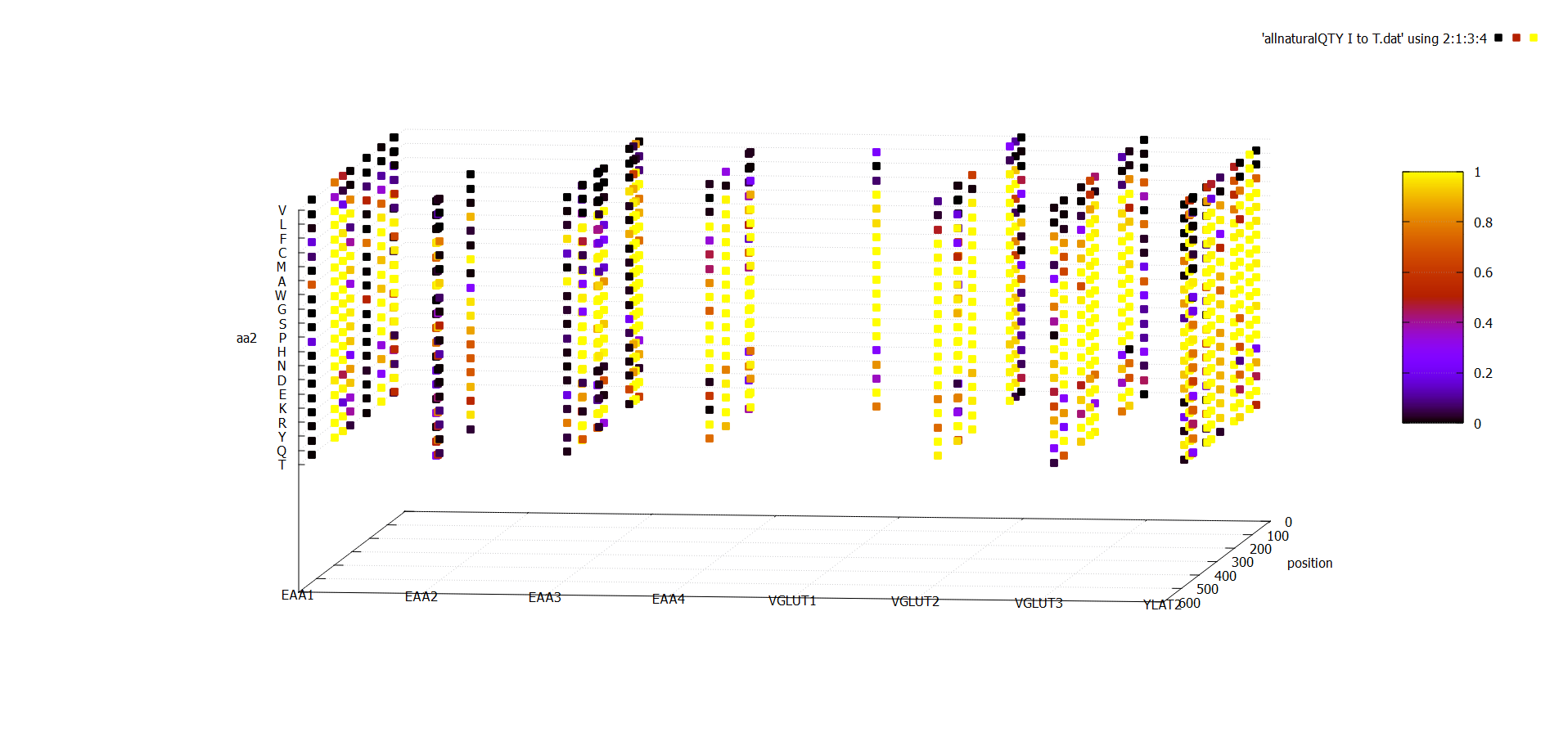


**Figure S8. Predicted effects of variations at the residue where natural I to T mutations observed.** The names of the transporters are listed on the x-axis. The z-axis indicates the position of the substitution within the native protein sequence, while the color scale represent the PolyPhen-2 predicted effect of the substitution, ranging from benign (0.0) to damaging (1.0). The y-axis indicates the second amino acid that replaced the wild-type (I) amino acid residue.


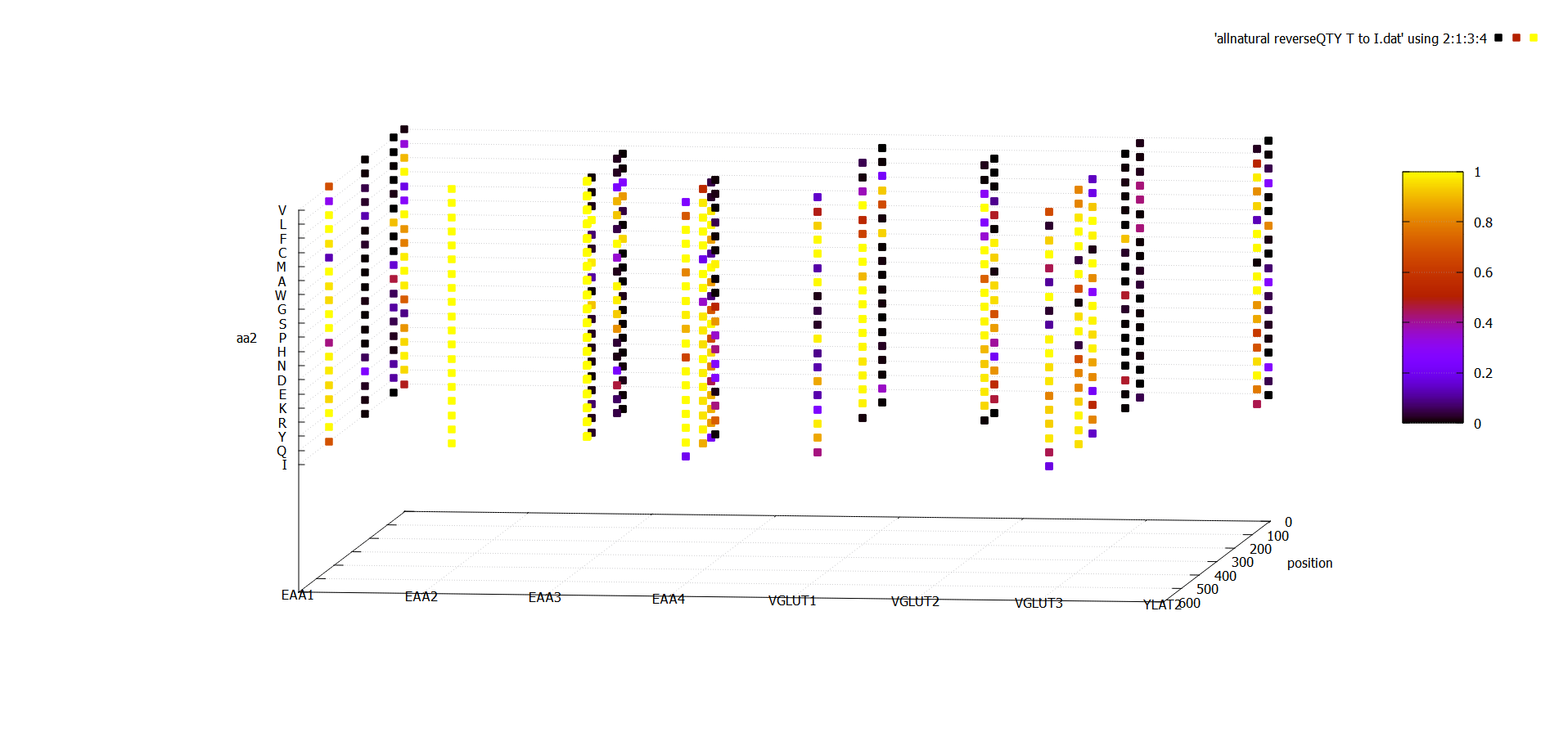


**Figure S9. Predicted effects of variations at the residue where natural T to I mutations observed.** The names of the transporters are listed on the x-axis. The z-axis indicates the position of the substitution within the native protein sequence, while the color scale represent the PolyPhen-2 predicted effect of the substitution, ranging from benign (0.0) to damaging (1.0). The y-axis indicates the second amino acid that replaced the wild-type (T) amino acid residue.


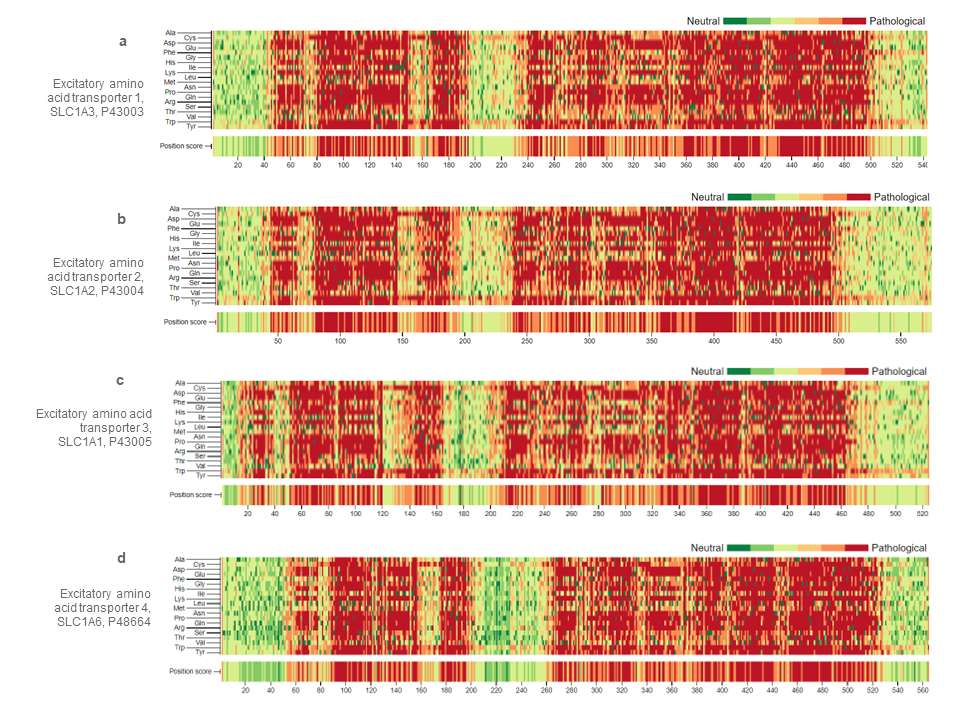


**Figure S10. Mutation visualizations of EAATs.** Pmut predicted pathologies of all possible mutations at each amino acid residue of 4 EAATs: EAA1 **(a)**, EAA2 **(b)**, EAA3 **(c)**, EAA4 **(d)**. The mutations are color-coded as green for neutral or red for pathological. The visualizations were obtained from the Pmut repository.


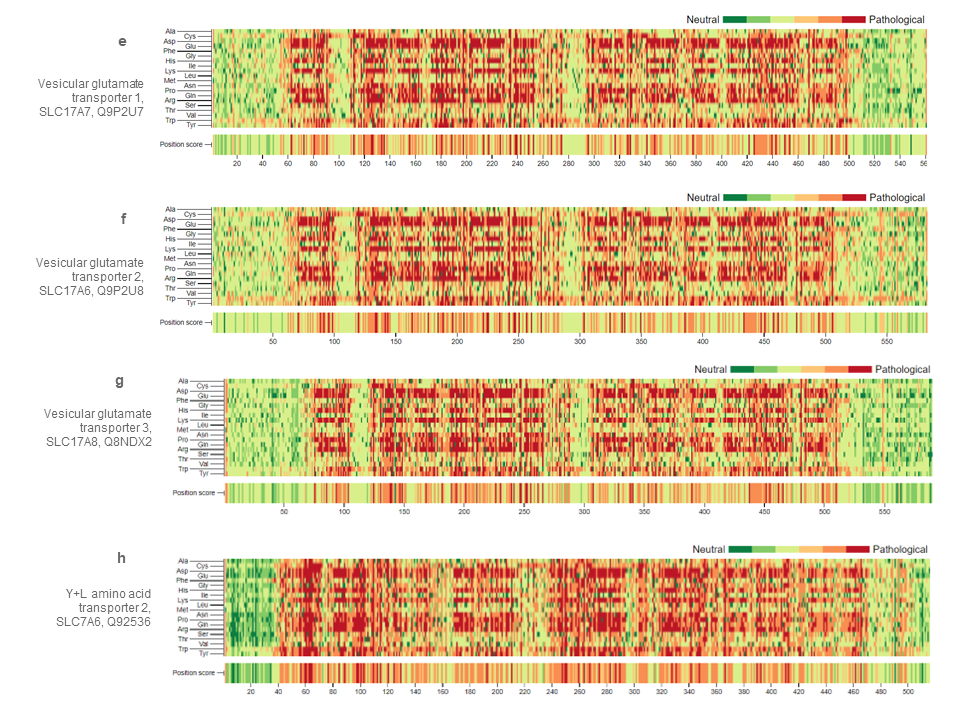


**Figure S11**. **Mutation visualizations of VGLUTs and YLAT2**. Pmut predicted pathologies of all possible mutations at each amino acid residue of 3 VGLUTs and YLAT2: VGLUT1 **(e)**, VGLUT2 **(f)**, VGLUT3 **(f)**, YLAT2 **(h)**. The mutations are color-coded as green for neutral or red for pathological. The visualizations were obtained from the Pmut repository.


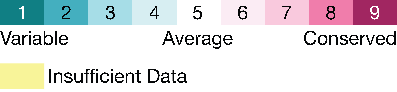

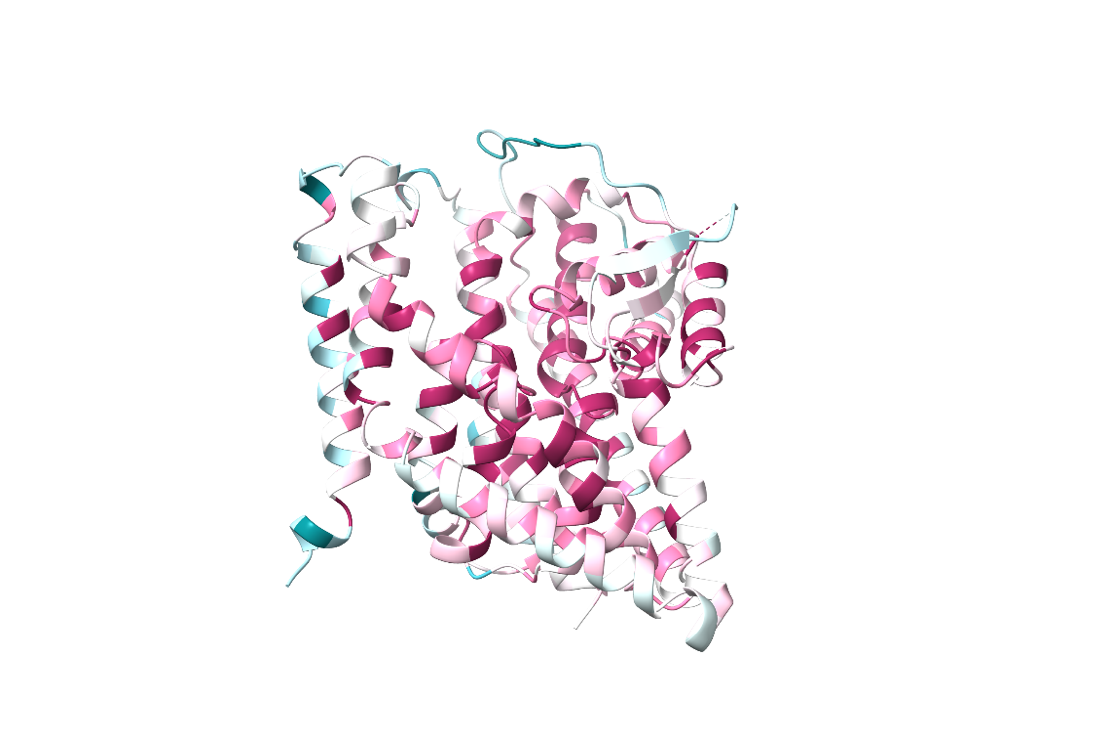

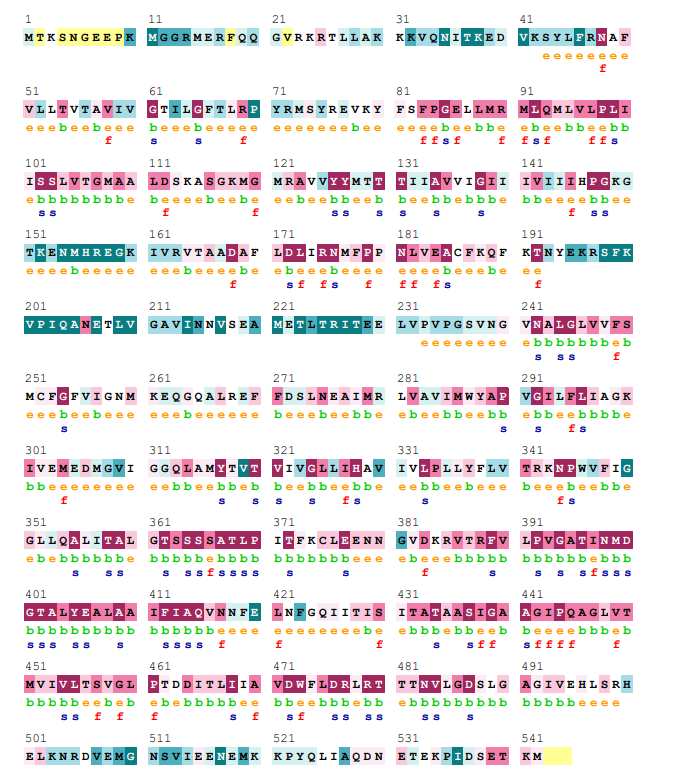


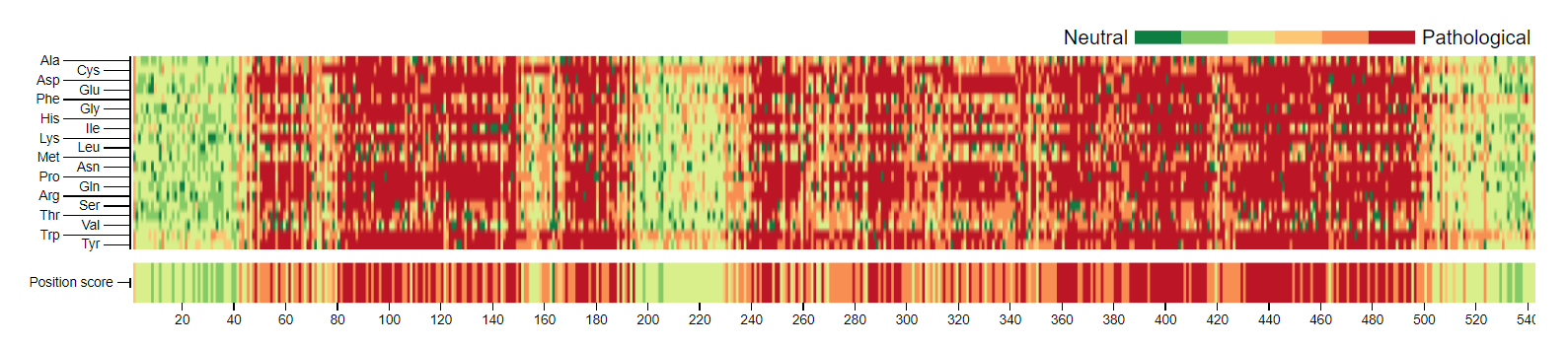


**Figure S12. EAA1 evolutionary conservation profiles and mutation visualizations.** Evolutionary conservation grades of each amino acid residue predicted by ConSurf server; visualized by the color-coding scheme of nine colors, ranging from turquoise (variable) through white (average) through burgundy (conserved) represents conservation grades 1 to 9, in order of increasing conservation (1= Variable, 5= Average, 9= Conserved). Conservation grades were calculated for the source amino acid sequence and the corresponding Alphafold2 predicted native structure. For clarity, the N- and C-termini and large loops, which are often not resolved in experimental structures, were deleted. At the bottom of the figure, the predicted pathologies of all possible EAA1 mutations displayed. The mutations are color-coded as green for neutral or red for pathological.


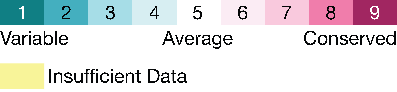

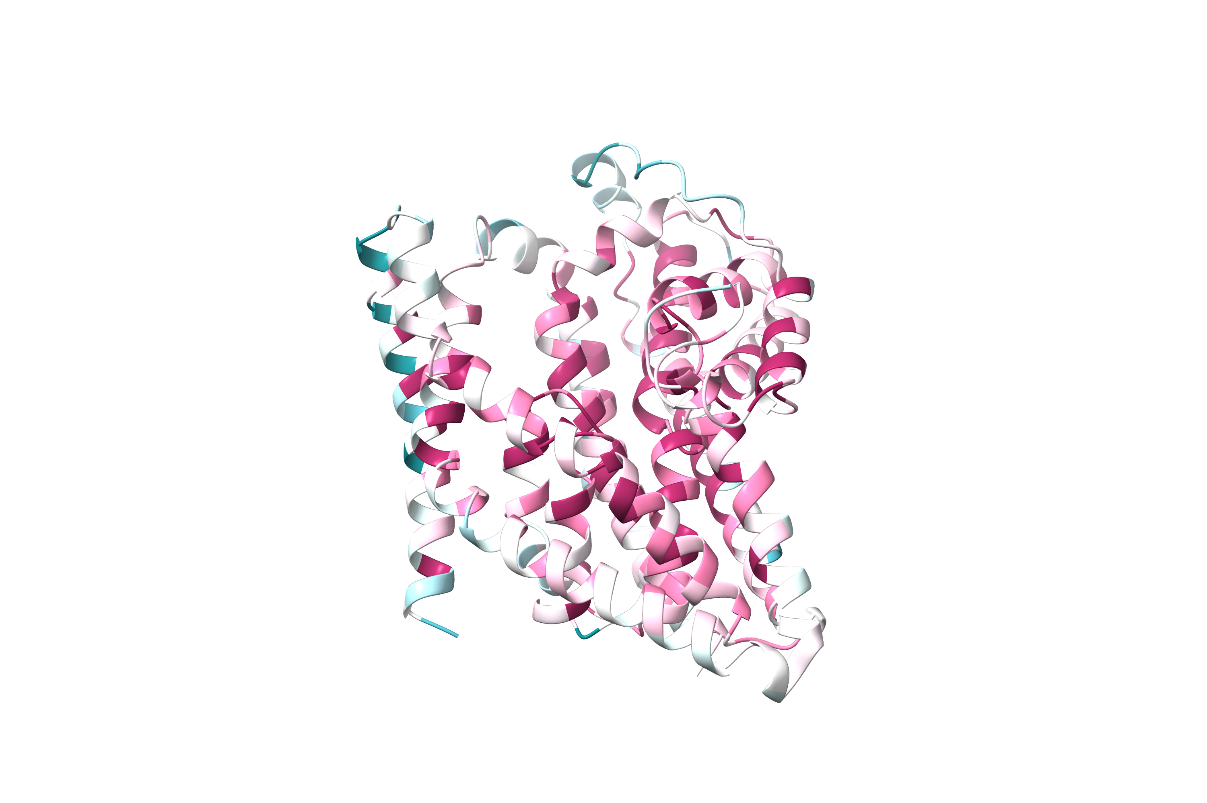

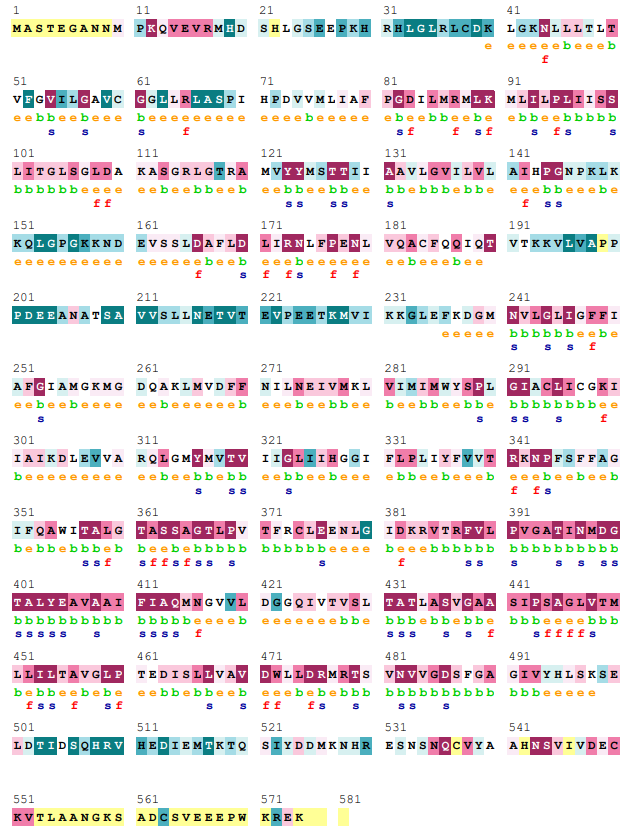


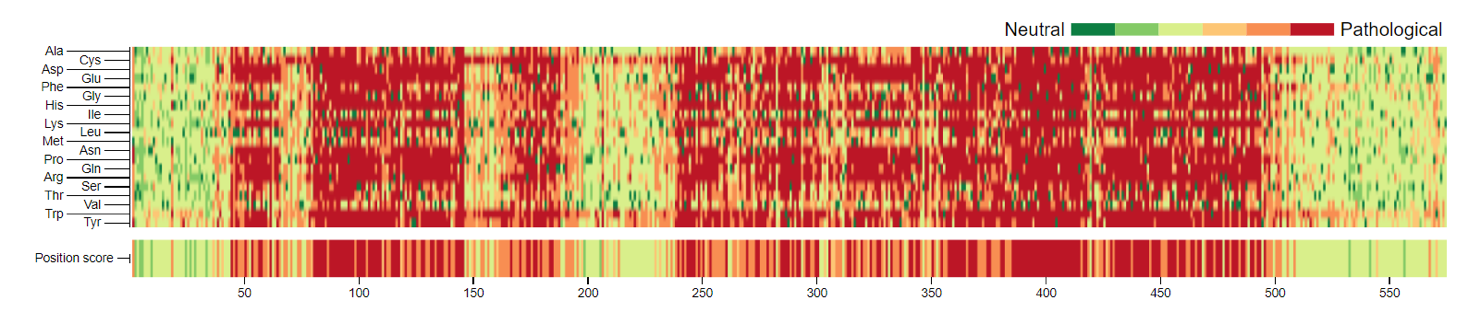


**Figure S13. EAA2 evolutionary conservation profiles and mutation visualizations.**

Evolutionary conservation grades of each amino acid residue predicted by ConSurf server; visualized by the color-coding scheme of nine colors, ranging from turquoise (variable) through white (average) through burgundy (conserved) represents conservation grades 1 to 9, in order of increasing conservation (1= Variable, 5= Average, 9= Conserved). Conservation grades were calculated for the source amino acid sequence and the corresponding Alphafold2 predicted native structure. For clarity, the N- and C-termini and large loops, which are often not resolved in experimental structures, were deleted. At the bottom of the figure, the predicted pathologies of all possible EAA2 mutations displayed. The mutations are color-coded as green for neutral or red for pathological.


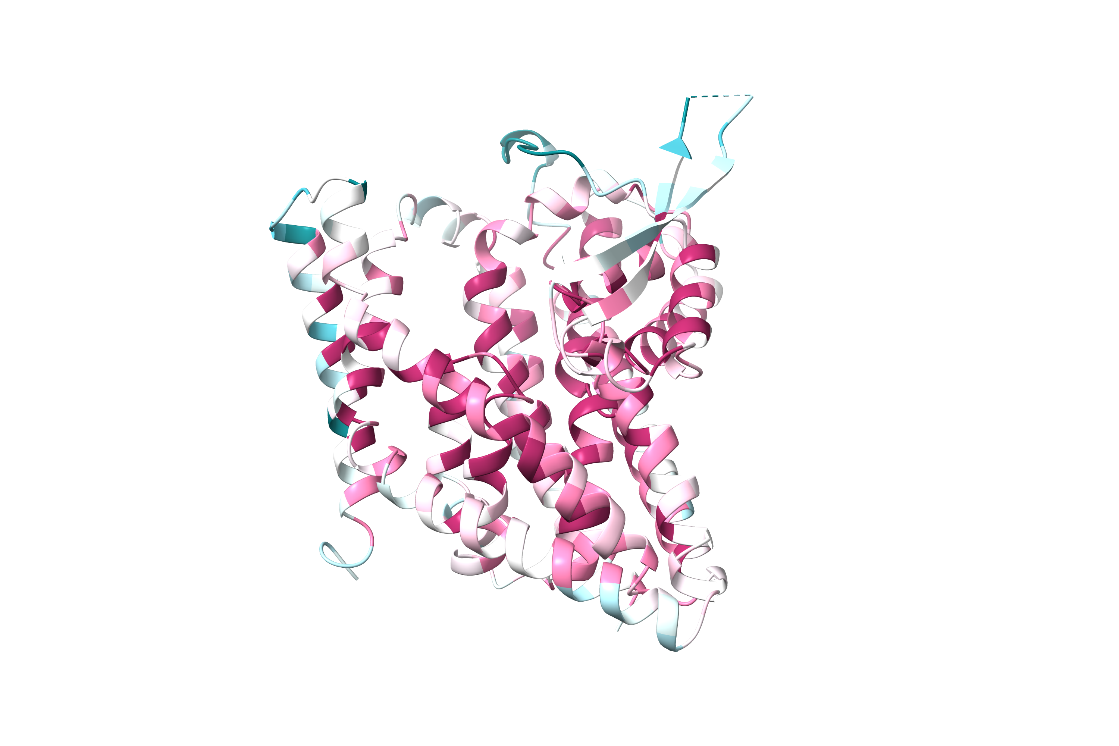

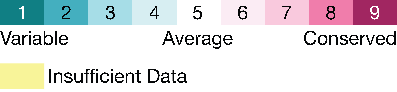

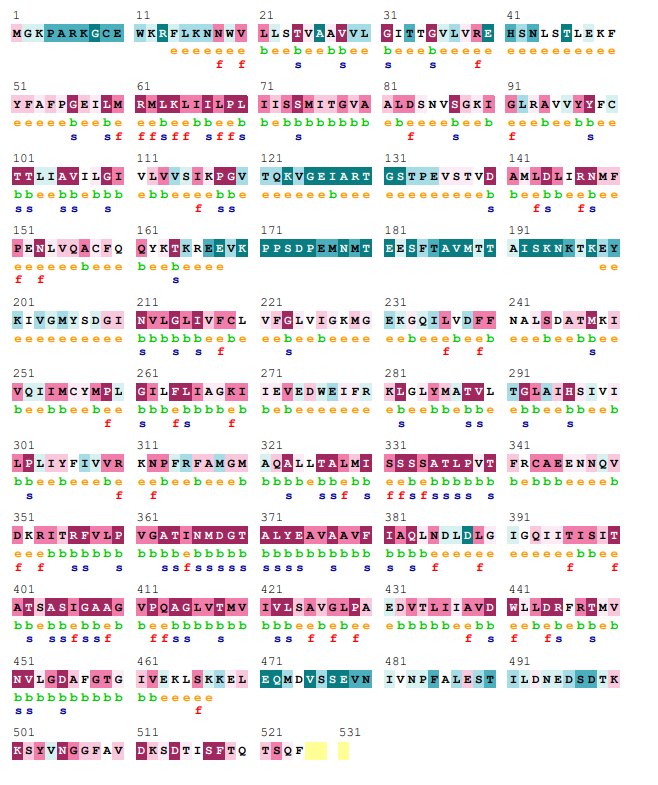


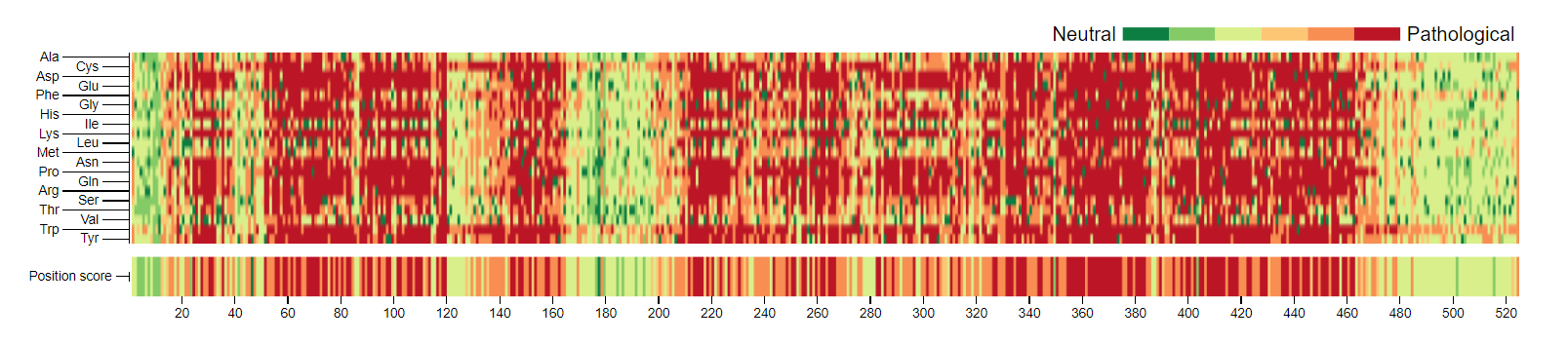


**Figure S14. EAA3 evolutionary conservation profiles and mutation visualizations.**

Evolutionary conservation grades of each amino acid residue predicted by ConSurf server; visualized by the color-coding scheme of nine colors, ranging from turquoise (variable) through white (average) through burgundy (conserved) represents conservation grades 1 to 9, in order of increasing conservation (1= Variable, 5= Average, 9= Conserved). Conservation grades were calculated for the source amino acid sequence and the corresponding Alphafold2 predicted native structure. For clarity, the N- and C-termini and large loops, which are often not resolved in experimental structures, were deleted. At the bottom of the figure, the predicted pathologies of all possible EAA3 mutations displayed. The mutations are color-coded as green for neutral or red for pathological.


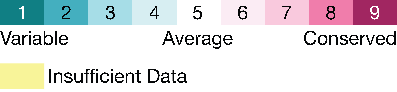

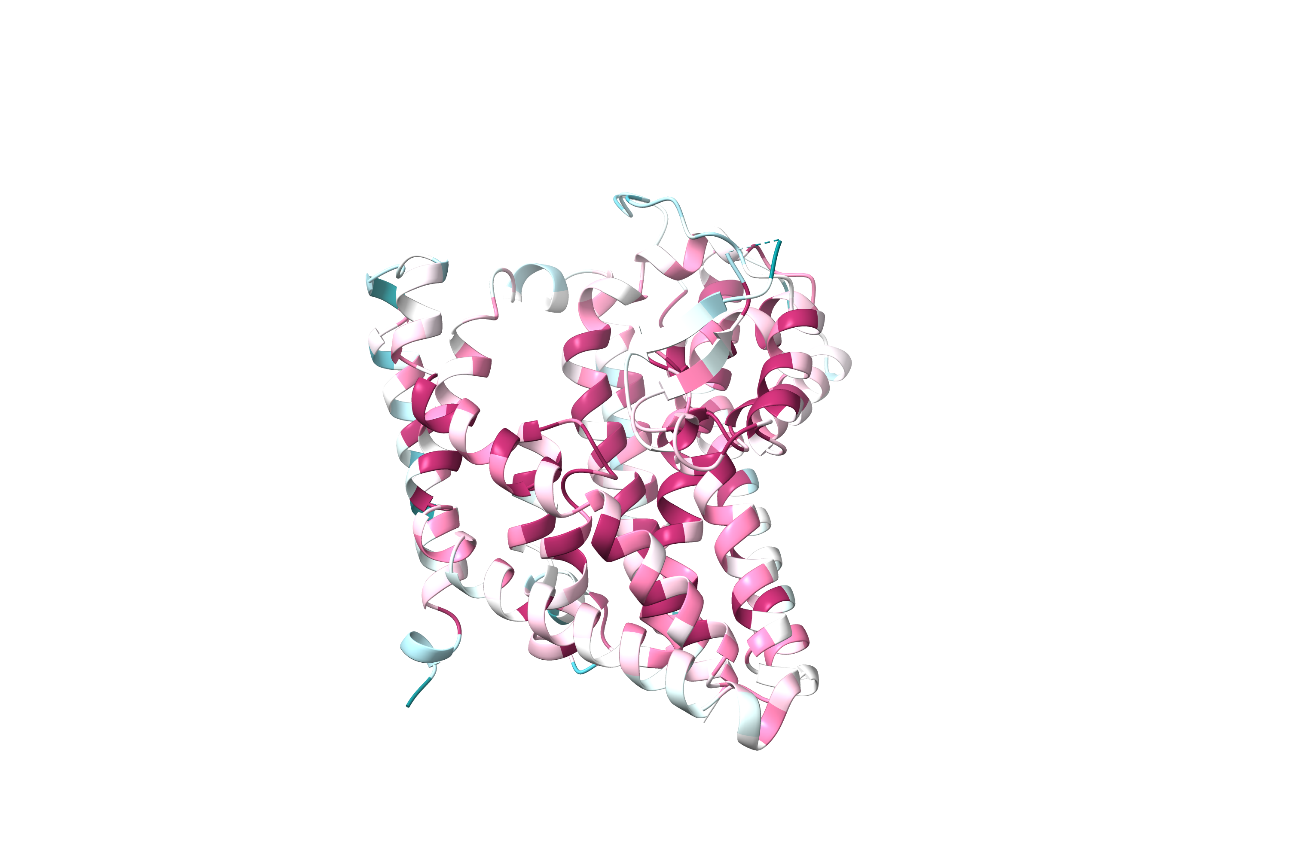

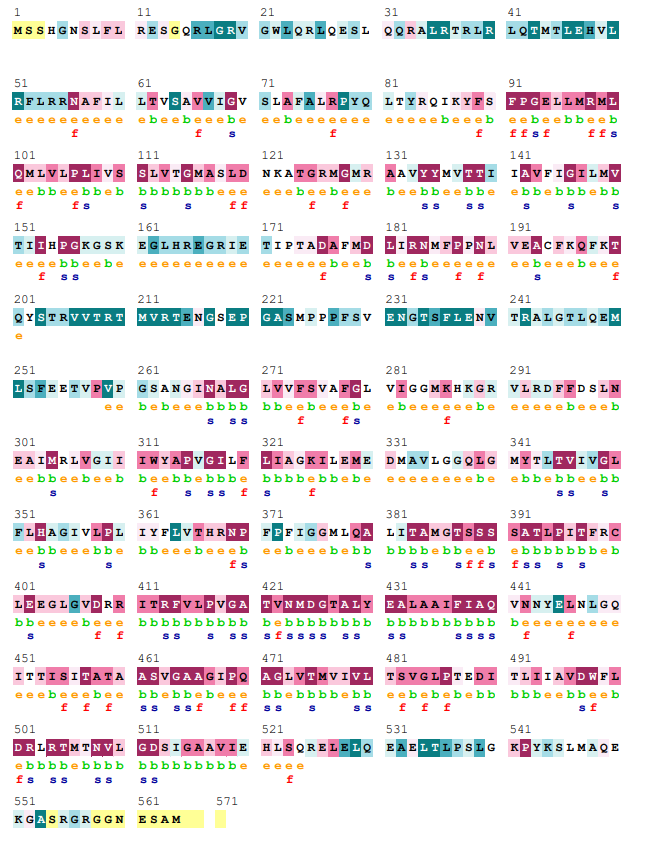


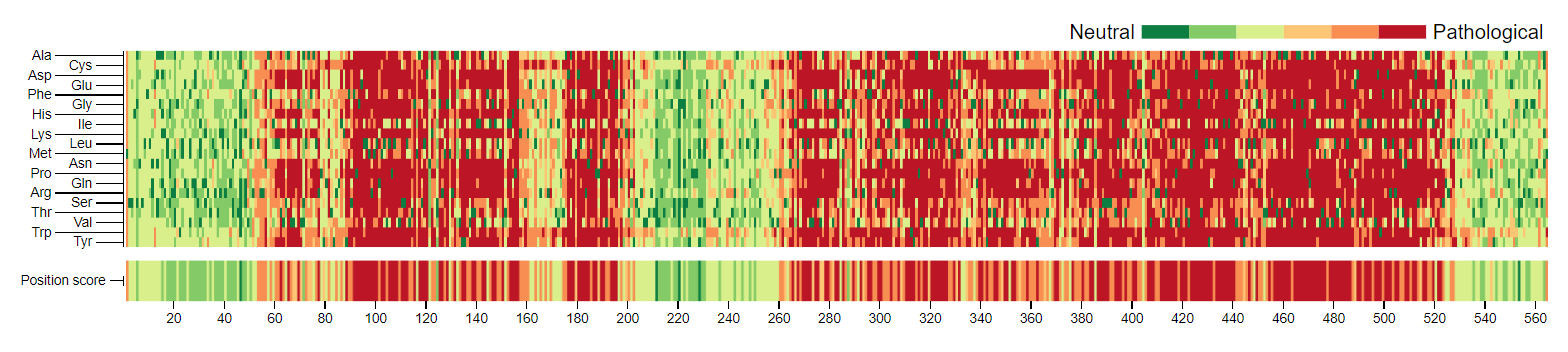


**Figure S15. EAA4 evolutionary conservation profiles and mutation visualizations.** Evolutionary conservation grades of each amino acid residue predicted by ConSurf server; visualized by the color-coding scheme of nine colors, ranging from turquoise (variable) through white (average) through burgundy (conserved) represents conservation grades 1 to 9, in order of increasing conservation (1= Variable, 5= Average, 9= Conserved). Conservation grades were calculated for the source amino acid sequence and the corresponding Alphafold2 predicted native structure. For clarity, the N- and C-termini and large loops, which are often not resolved in experimental structures, were deleted. At the bottom of the figure, the predicted pathologies of all possible EAA4 mutations displayed. The mutations are color-coded as green for neutral or red for pathological.


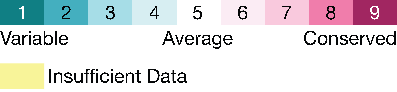

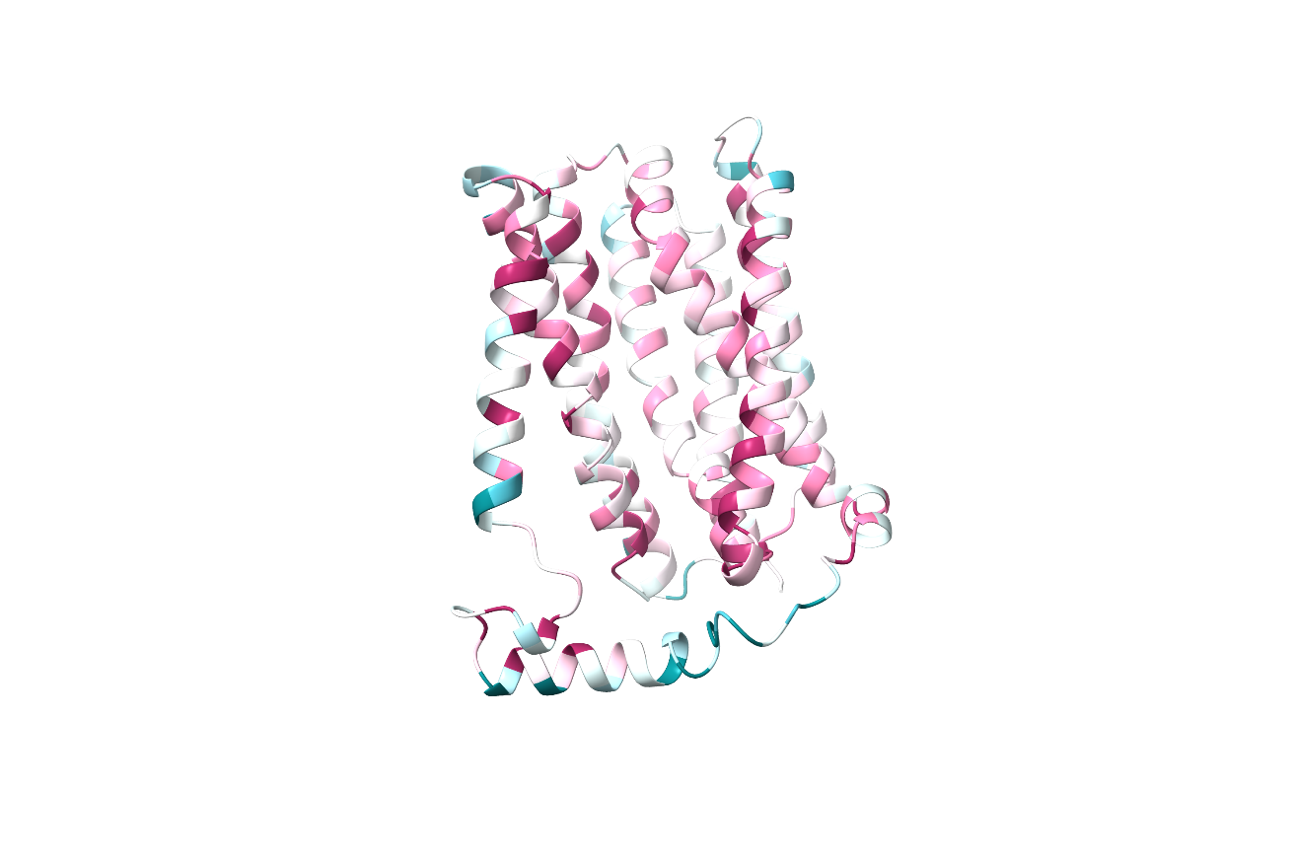

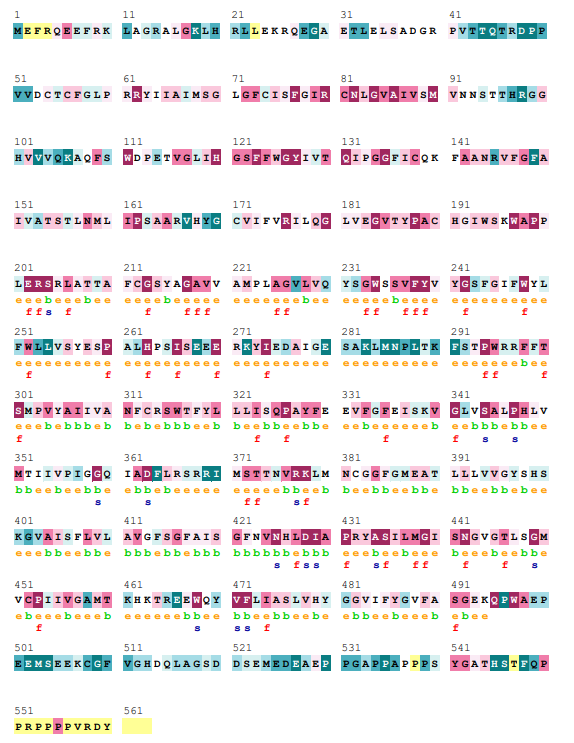


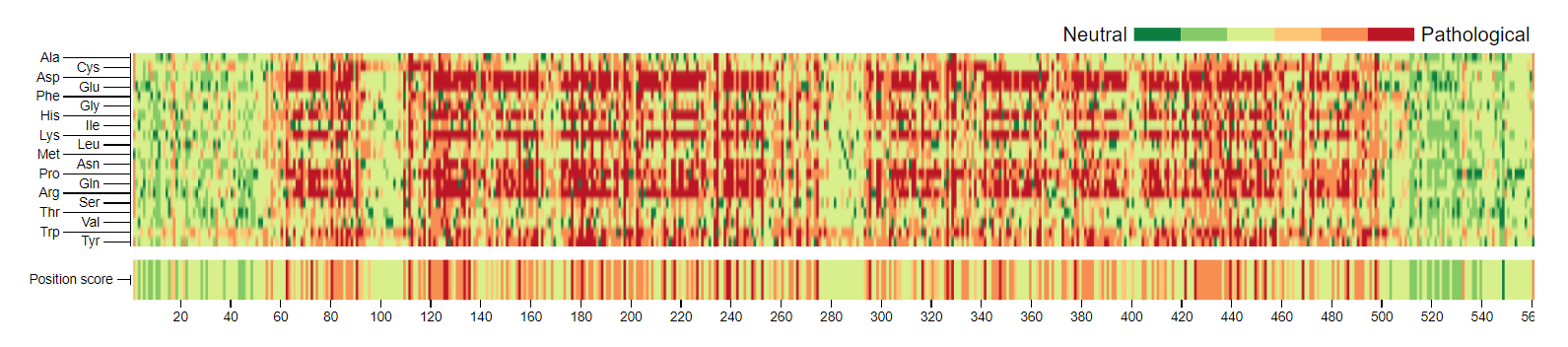


**Figure S16. VGLUT1 evolutionary conservation profiles and mutation visualizations.** Evolutionary conservation grades of each amino acid residue predicted by ConSurf server; visualized by the color-coding scheme of nine colors, ranging from turquoise (variable) through white (average) through burgundy (conserved) represents conservation grades 1 to 9, in order of increasing conservation (1= Variable, 5= Average, 9= Conserved). Conservation grades were calculated for the source amino acid sequence and the corresponding Alphafold2 predicted native structure. For clarity, the N- and C-termini and large loops, which are often not resolved in experimental structures, were deleted. At the bottom of the figure, the predicted pathologies of all possible VGLUT1 mutations displayed. The mutations are color-coded as green for neutral or red for pathological.


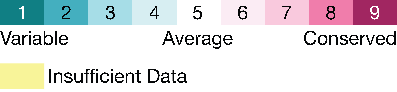

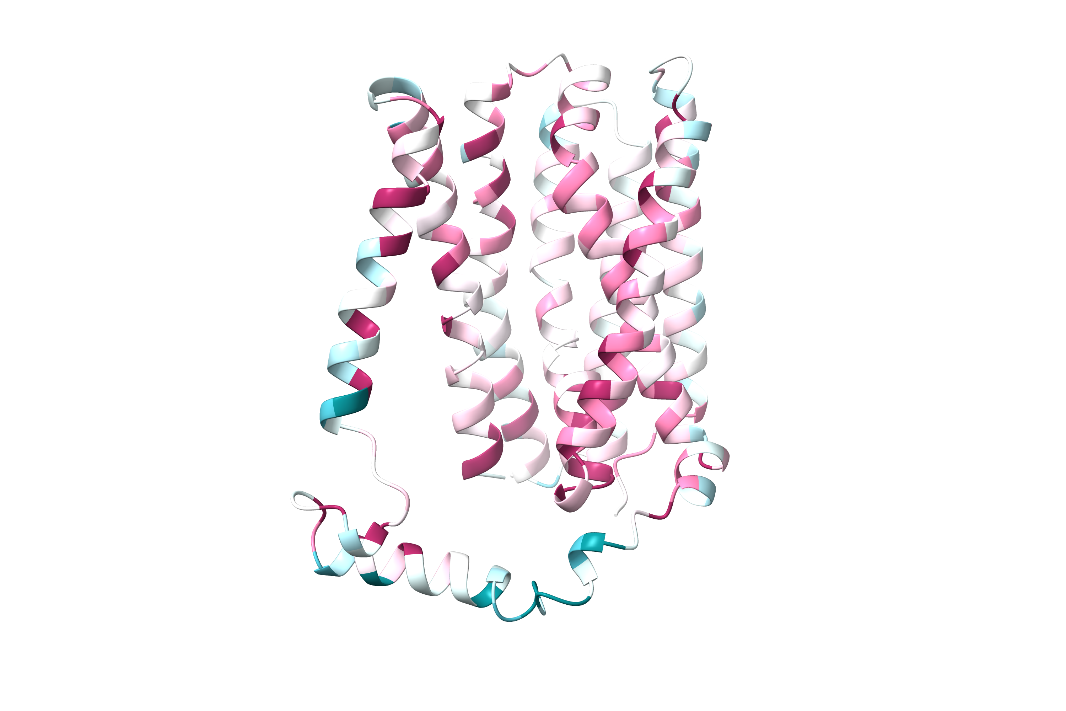

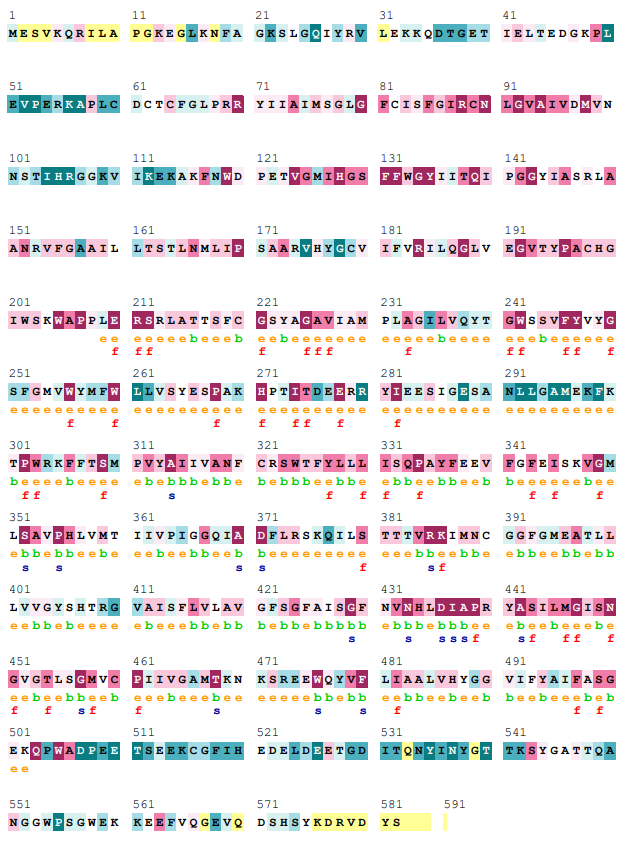


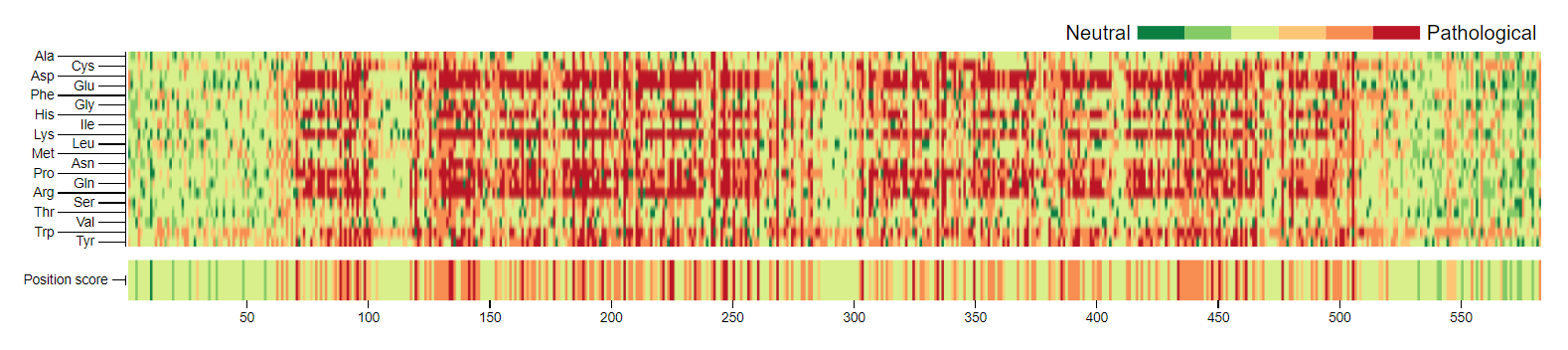


**Figure S17. VGLUT2 evolutionary conservation profiles and mutation visualizations.** Evolutionary conservation grades of each amino acid residue predicted by ConSurf server; visualized by the color-coding scheme of nine colors, ranging from turquoise (variable) through white (average) through burgundy (conserved) represents conservation grades 1 to 9, in order of increasing conservation (1= Variable, 5= Average, 9= Conserved). Conservation grades were calculated for the source amino acid sequence and the corresponding Alphafold2 predicted native structure. For clarity, the N- and C-termini and large loops, which are often not resolved in experimental structures, were deleted. At the bottom of the figure, the predicted pathologies of all possible VGLUT2 mutations displayed. The mutations are color-coded as green for neutral or red for pathological.


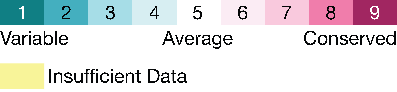

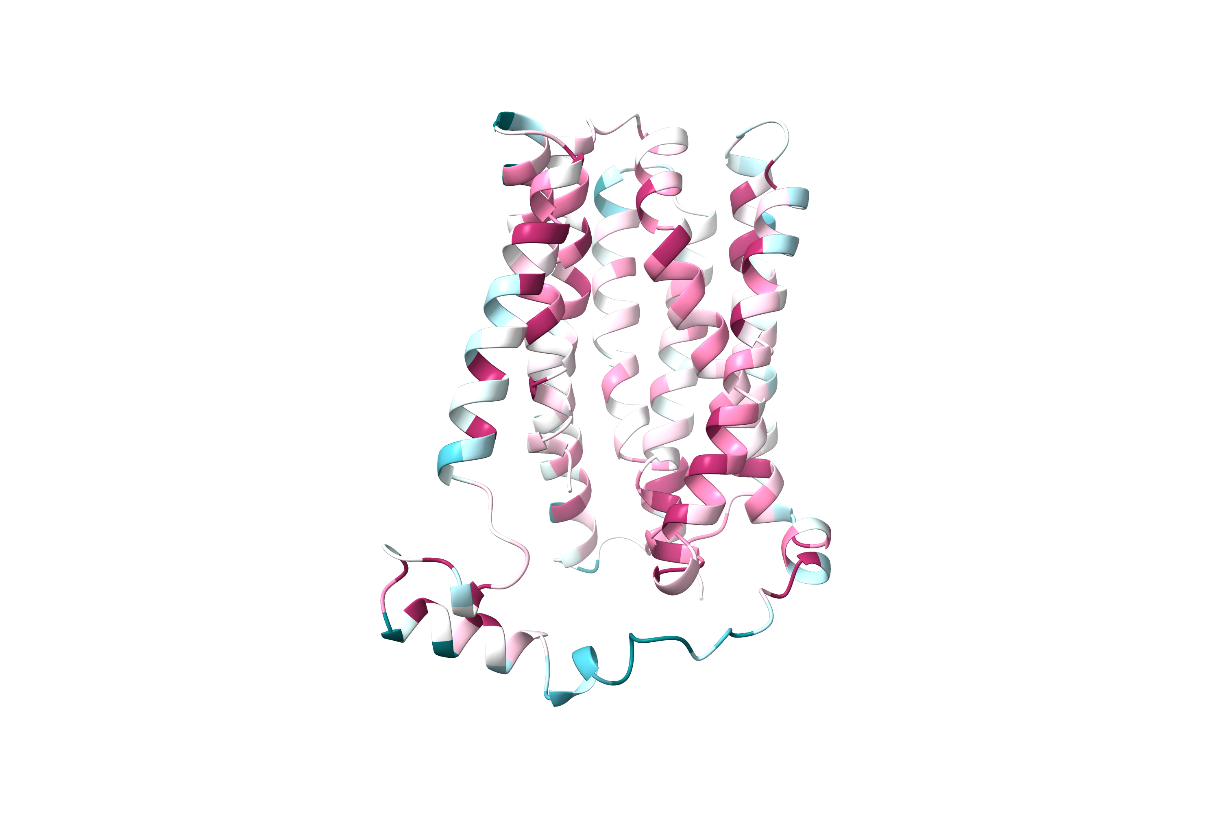

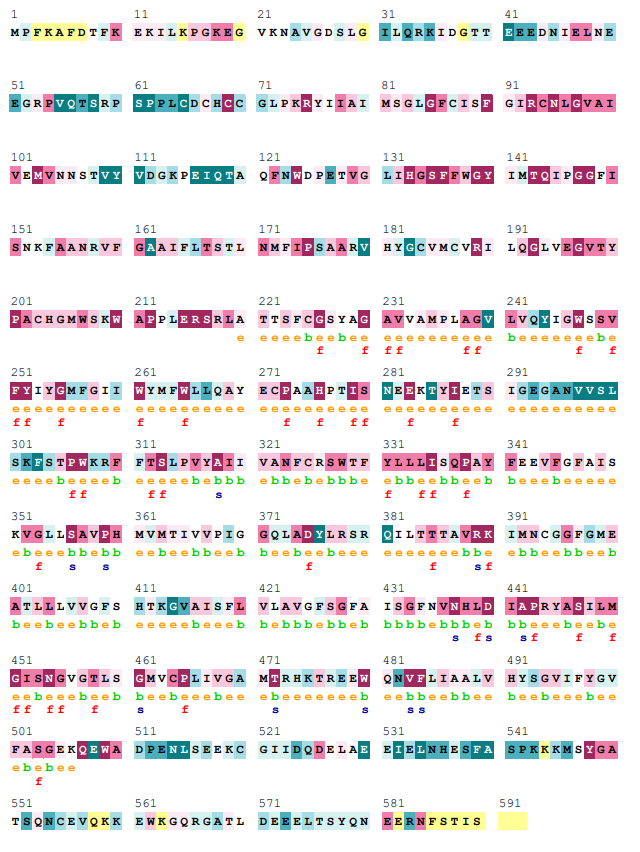

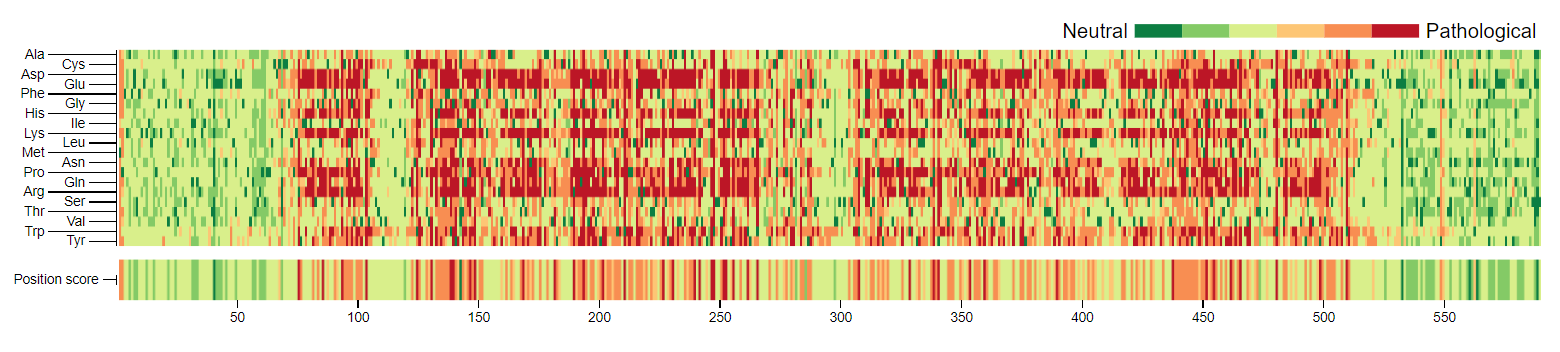


**Figure S18. VGLUT3 evolutionary conservation profiles and mutation visualizations**. Evolutionary conservation grades of each amino acid residue predicted by ConSurf server; visualized by the color-coding scheme of nine colors, ranging from turquoise (variable) through white (average) through burgundy (conserved) represents conservation grades 1 to 9, in order of increasing conservation (1= Variable, 5= Average, 9= Conserved). Conservation grades were calculated for the source amino acid sequence and the corresponding Alphafold2 predicted native structure. For clarity, the N- and C-termini and large loops, which are often not resolved in experimental structures, were deleted. At the bottom of the figure, the predicted pathologies of all possible VGLUT3 mutations displayed. The mutations are color-coded as green for neutral or red for pathological.


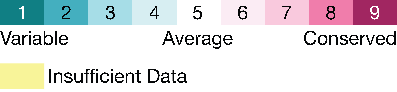

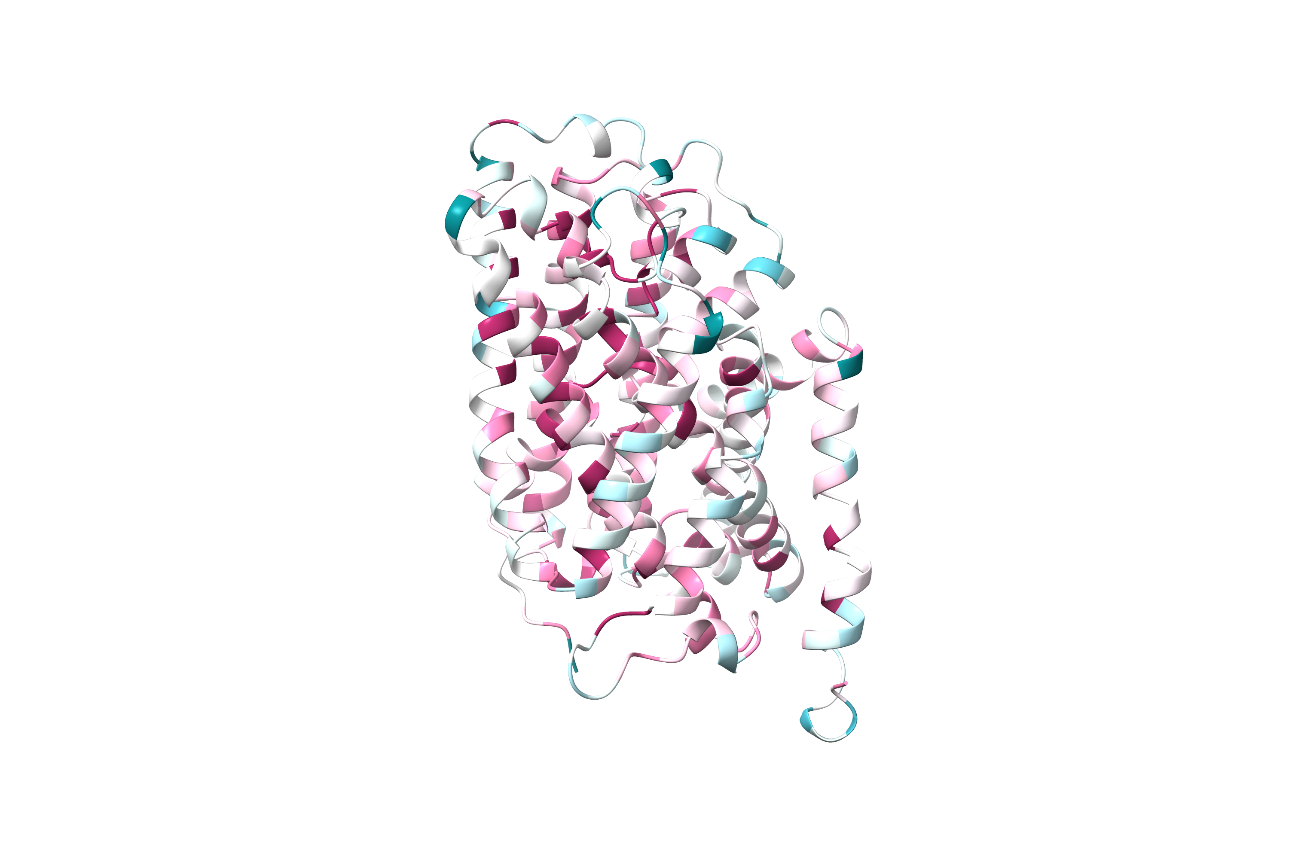

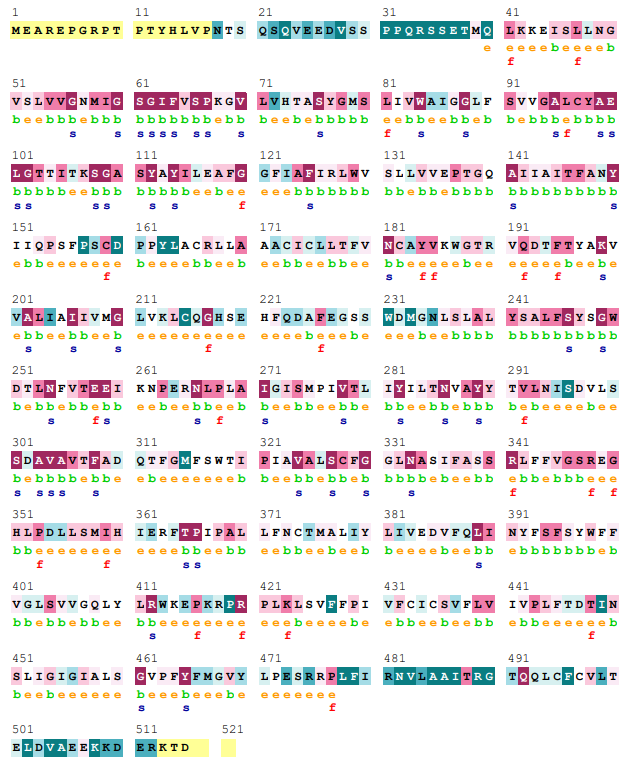


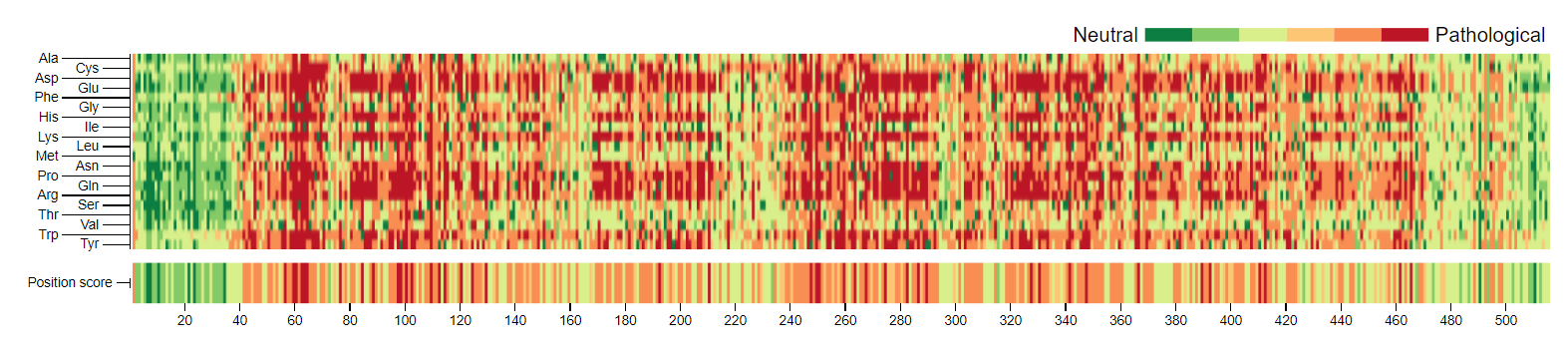


**Figure S19**. **YLAT2 evolutionary conservation profiles and mutation visualizations.** Evolutionary conservation grades of each amino acid residue predicted by ConSurf server; visualized by the color-coding scheme of nine colors, ranging from turquoise (variable) through white (average) through burgundy (conserved) represents conservation grades 1 to 9, in order of increasing conservation (1= Variable, 5= Average, 9= Conserved). Conservation grades were calculated for the source amino acid sequence and the corresponding Alphafold2 predicted native structure. For clarity, the N- and C-termini and large loops, which are often not resolved in experimental structures, were deleted. At the bottom of the figure, the predicted pathologies of all possible YLAT2 mutations displayed. The mutations are color-coded as green for neutral or red for pathological.


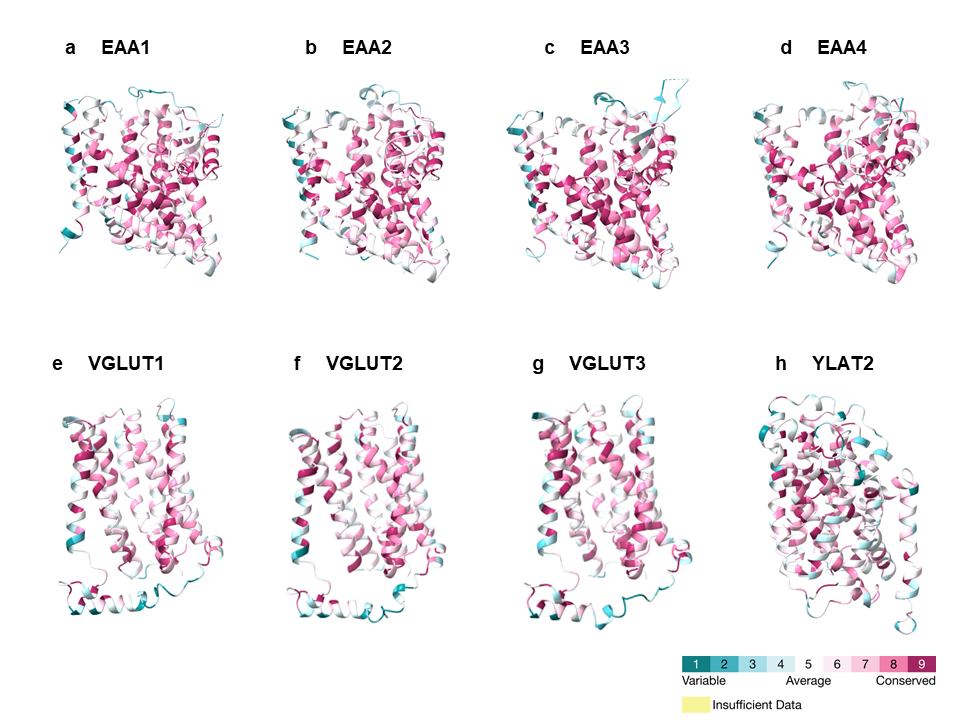


**Figure S20**. **Evolutionary conservation profiles of the 8 native glutamate transporters** **that were predicted by AlphaFold2.** The predicted native structures and their residues colored by evolutionary conservation grades. Average conservation grades for each structure are **a** 6.5 for EAA1 **b** 6.5 for EAA2, **c** 6.6 for EAA3 **d,** 6.7 for EAA4, **e** 6.0 for VGLUT1, **f** 6.1 for VGLUT2, **g** 6.1 for VGLUT3, **h** 6.1 for YLAT2. For clarity, N- and C- termini and large loops are deleted.


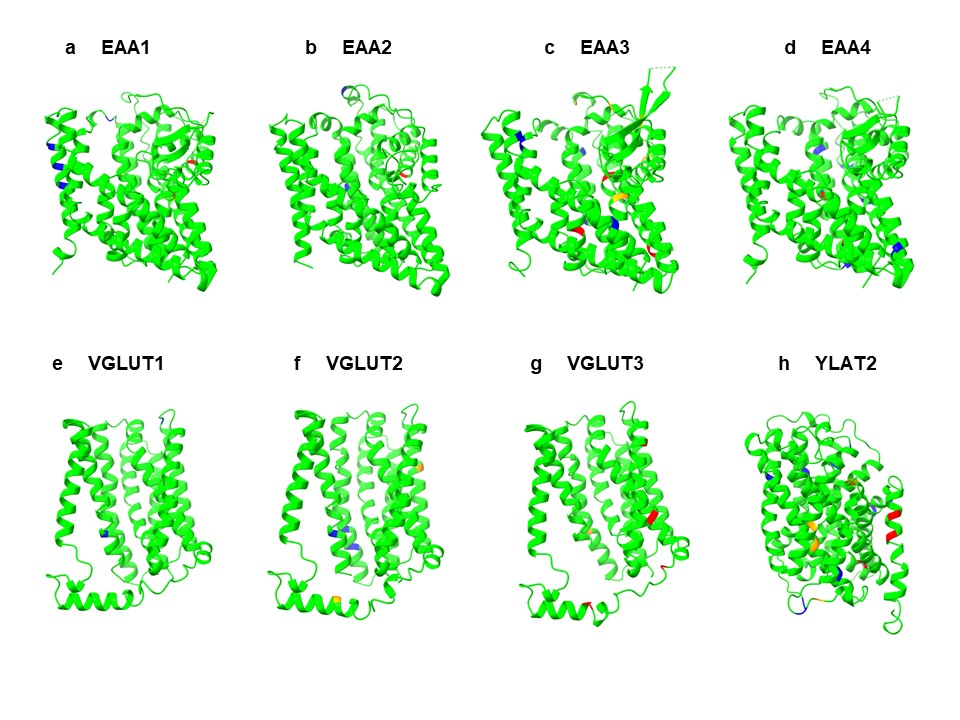


**Figure S21**. **Natural mutations of QTY-code and rQTY code**. The native structures (green) and predicted effects of QTY and reverse QTY mutations shown as colored residues. Blue = benign, orange = possibly damaging with low confidence, red= damaging with high confidence. **a** EAA1, **b** EAA2, **c** EAA3, **d** EAA4, **e** VGluT1, **f** VGluT2, **g** VGluT3, **h** YLAT2. For clarity, N- and C termini and large loops are deleted.

**Figure S22. Enlarged panel a-h of Figure 1.** The protein sequences of each glutamate transporter are now clearly visible. The QTY variant sequences are below the native protein sequences. The QTY amino acid substitution changes are colored in red. Other colour code: Yellow line-intracellular, Blue wave-transmembrane helices, Pinkish line-extracellular, Green line-peripheral domains and hairpin loops.


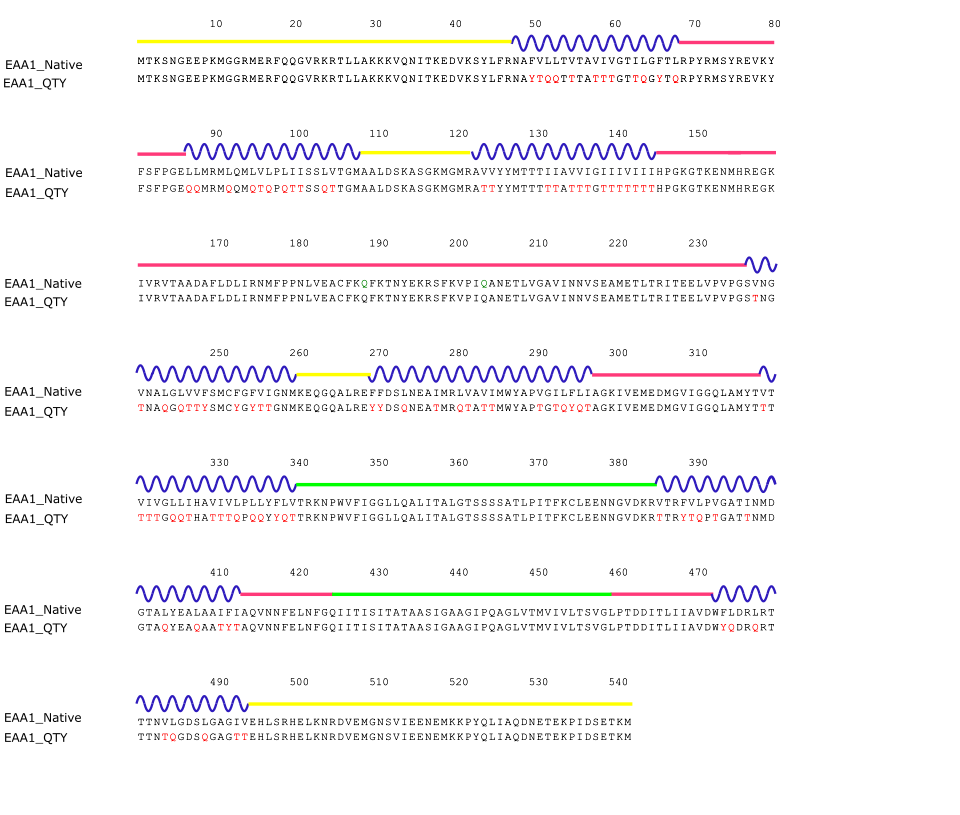


**a**, EAA1


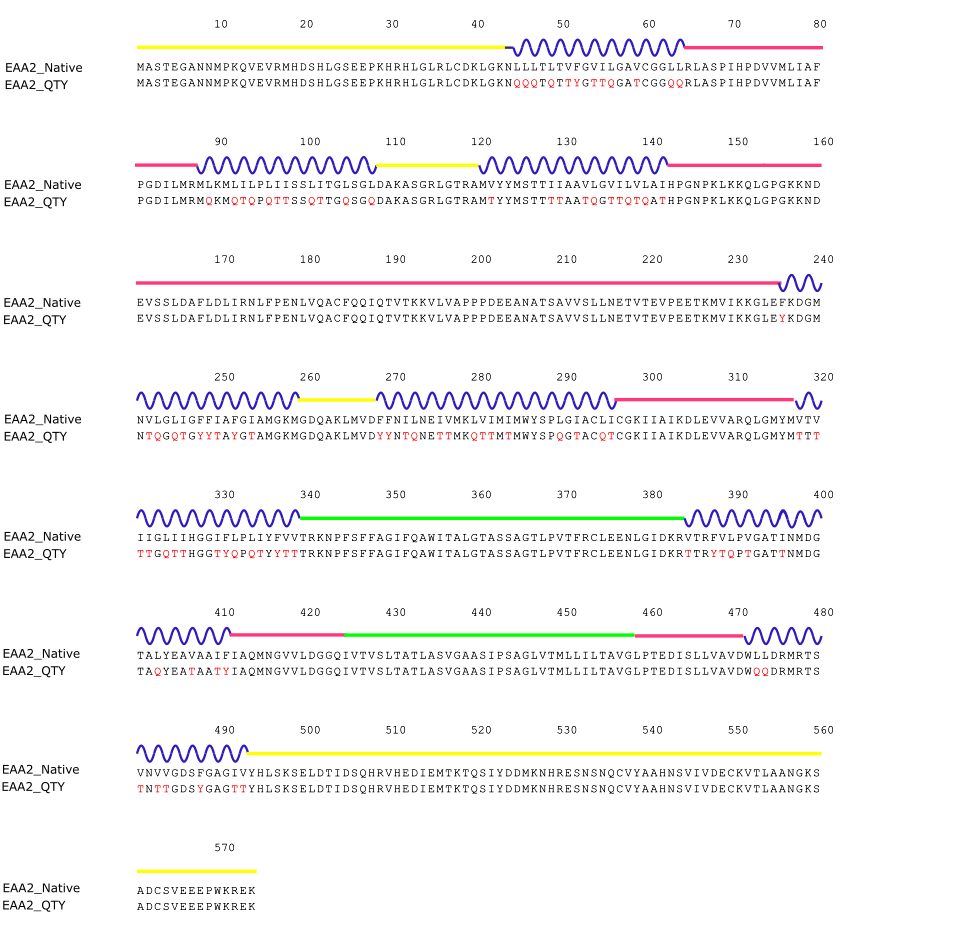


**b**, EAA2


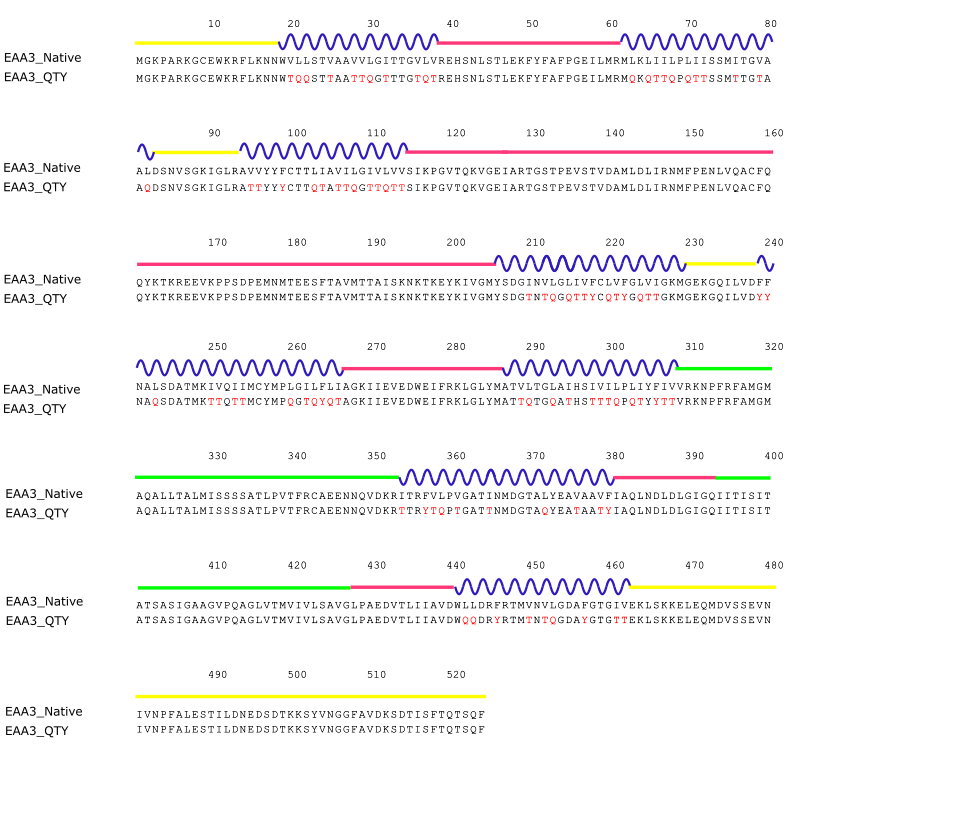


**c**, EAA3


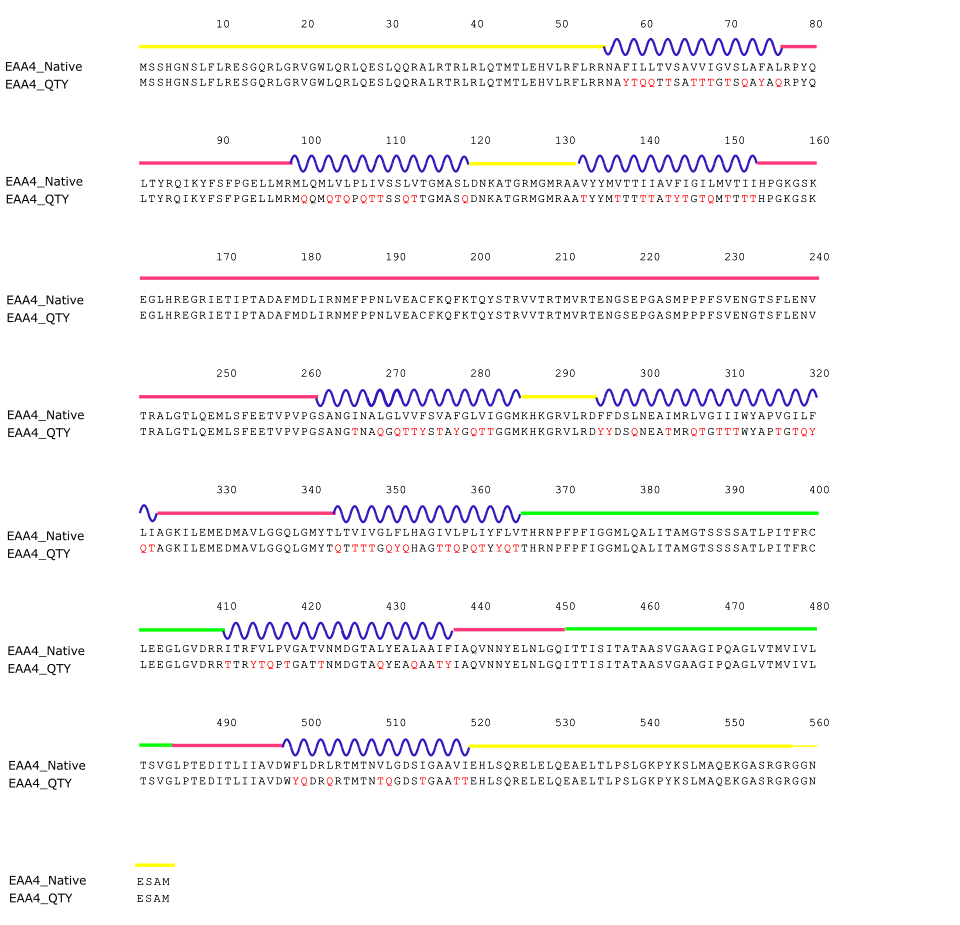


**d**, EAA4


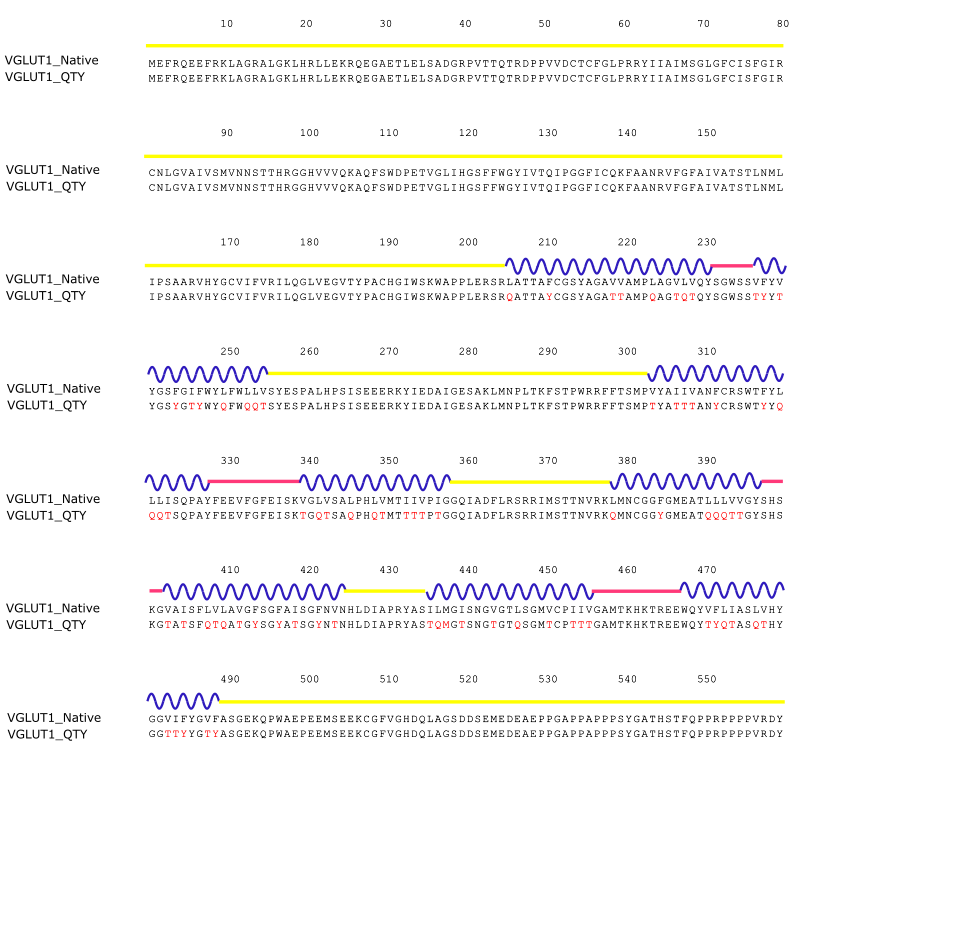


**e**, VGLUT1


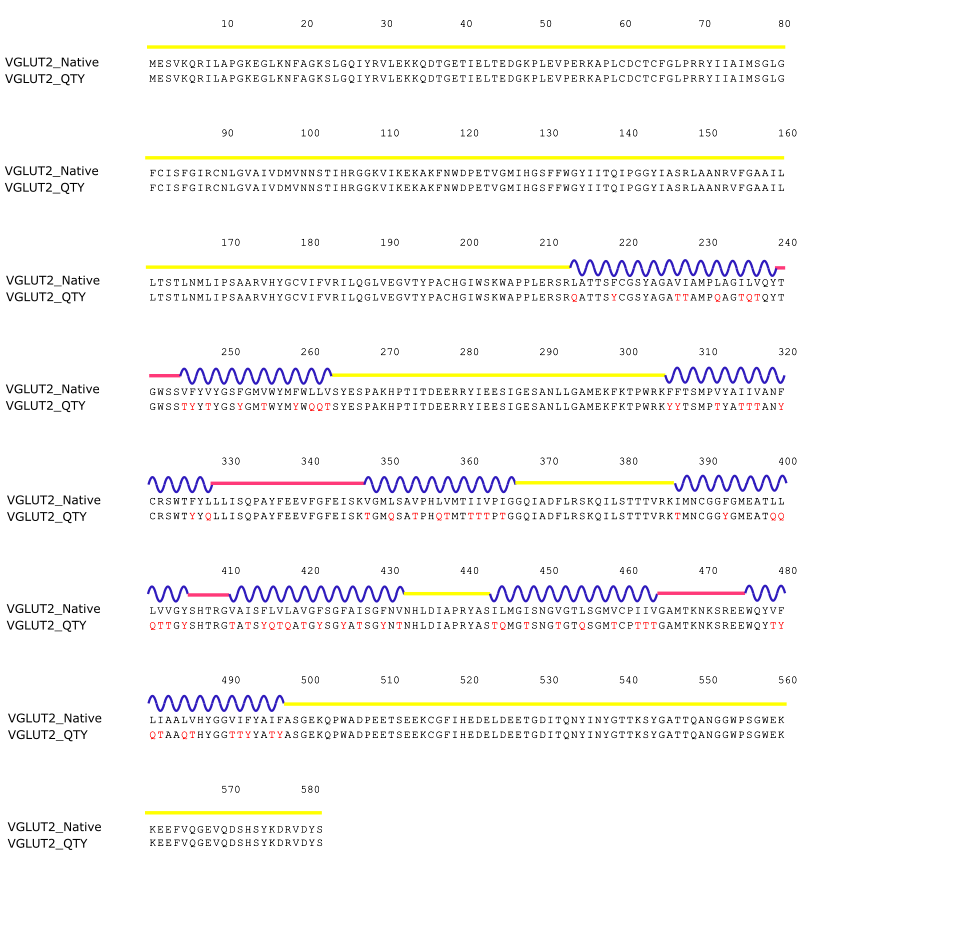


**f**, VGLUT2


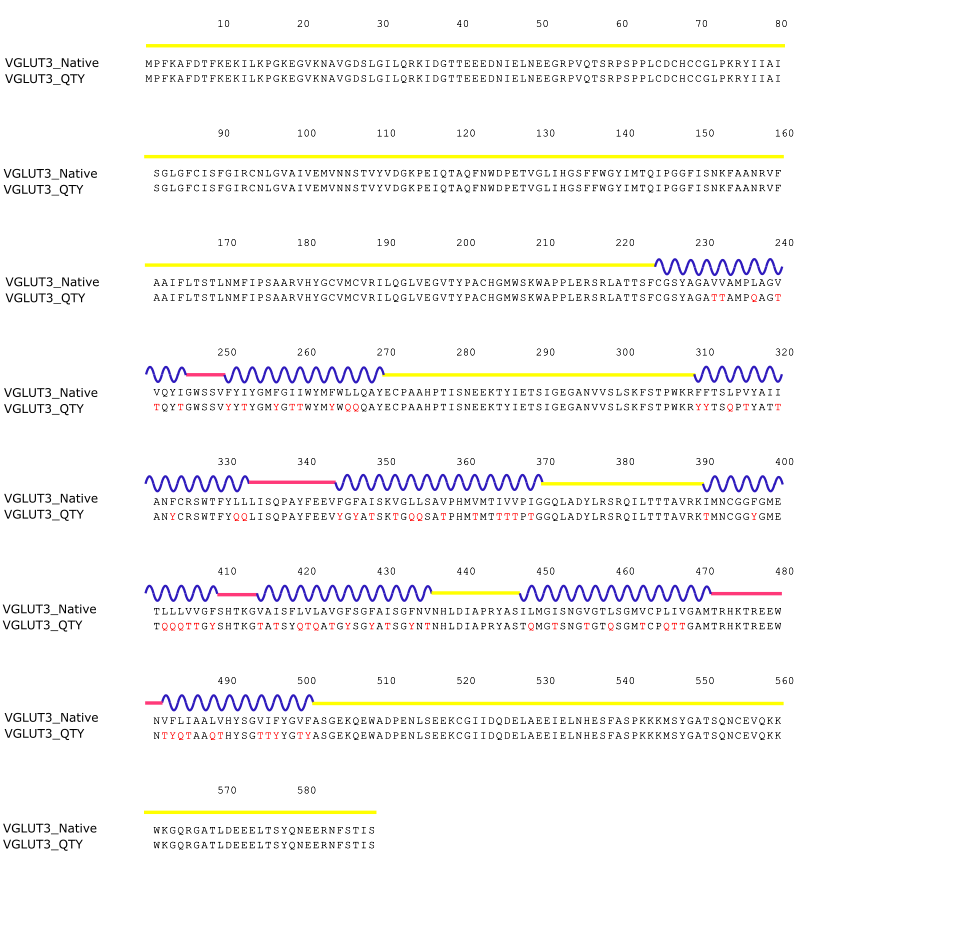


**g**, VGLUT3


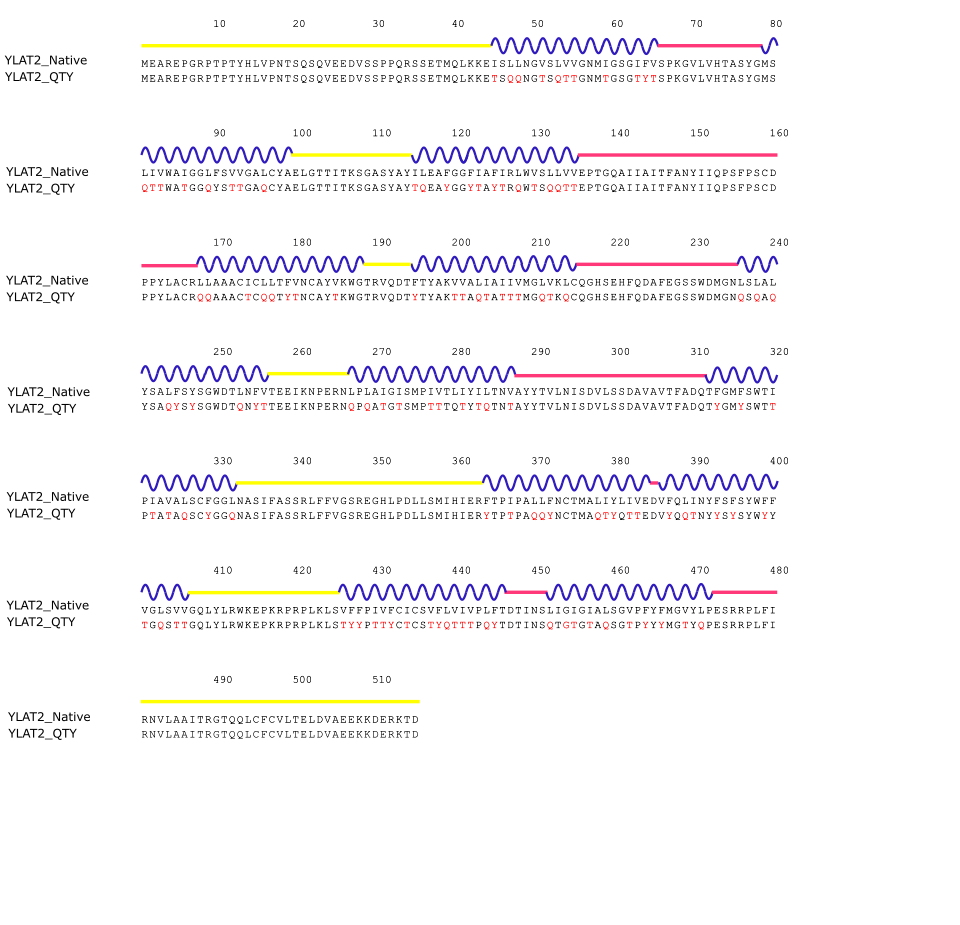


**h**, YLAT2
